# Field-crop transcriptome models are enhanced by measurements in systematically controlled environments

**DOI:** 10.1186/s13059-025-03690-8

**Published:** 2025-07-28

**Authors:** Yoichi Hashida, Daisuke Kyogoku, Suguru E. Tanaka, Naoya Mori, Takanari Tanabata, Hiroyuki Watanabe, Atsushi J. Nagano

**Affiliations:** 1 Faculty of Agriculture, Takasaki University of Health and Welfare, Takasaki, Gunma Japan; 2 Museum of Nature and Human Activities, Sanda, Hyogo Japan; 3 Faculty of Science, Nara Women’s University, Nara, Nara Japan; 4 Research Institute for Food and Agriculture, Ryukoku University, Otsu, Shiga Japan; 5 Biosystems & Biofunctions Research Center, Research Institute, Tamagawa University, Machida, Tokyo, Japan; 6 Facility for Genome Informatics, Kazusa DNA Research Institute, Kisarazu, Chiba, Japan; 7 Graduate School of Science and Technology, Chitose Institute of Science and Technology, Chitose, Hokkaido Japan; 8 Department of Advanced Food Sciences, Faculty of Agriculture, Tamagawa University, Machida, Tokyo, Japan; 9 Faculty of Agriculture, Ryukoku University, Yokotani 1-5, Seta Ohe-Cho, Otsu, Shiga 520-2194 Japan; 10 Institute for Advanced Biosciences, Keio University, Tsuruoka, Yamagata Japan; 11 Bioscience and Biotechnology Center, Nagoya University, Nagoya, Aichi Japan

**Keywords:** Rice, Field, Growth chamber, RNA-Seq, Statistical modeling, Transcriptome

## Abstract

**Background:**

Plants in the field respond to seasonal and diel changes in various environmental factors such as irradiance and temperature. We previously developed a statistical model that predicts rice gene expression from the meteorological data and identified the environmental factors regulating each gene. However, since irradiance and temperature—the two most critical environmental factors—are correlated in the field, it remains difficult to distinguish their roles in gene expression regulation.

**Results:**

We show that transcriptome dynamics in the field are predominantly regulated by irradiance, by the modeling involving diel transcriptome data from the 73 controlled conditions where irradiance and temperature are independently varied. The model’s prediction performance is substantially high when trained using field and controlled conditions data.

**Conclusions:**

Our results highlight the utility of a systematic sampling approach under controlled environments to understand the mechanism of plant environmental response and to improve transcriptome prediction under field environments.

**Supplementary Information:**

The online version contains supplementary material available at 10.1186/s13059-025-03690-8.

## Background

Plants in nature and crops in agricultural fields adapt to seasonal and diel changes in various environmental factors such as irradiance and temperature. Since these environmental factors fluctuate at fine spatiotemporal scales, plants must extract essential information from the field environment for their survival and respond to them accordingly. Understanding how plants sense and respond to these environmental variations is crucial for elucidating their adaptation mechanisms and increasing crop production [1].

Although controlled environments such as glasshouses or growth chambers (GC) are commonly used in plant environmental response studies, findings obtained in such studies have not fully captured the complexities of plant responses to field conditions [2]. To address this issue, omics analysis has emerged as a promising way to study plant environmental responses to the field [3–5]. In particular, seasonal and diel transcriptome analysis has proven effective in elucidating how plants respond to different environmental stimuli in the field [6–16].

The complementary approach is to mimic fluctuating field conditions in controlled environments. This approach has the advantages of repeatability and varying only environmental factors in interest [17–21]. Such studies have provided insight into several aspects of plant physiology, including photosynthesis [22–24], flowering [25–27], and metabolic regulation [28, 29].

Statistical modeling approaches have effectively extracted meaningful insights from noisy transcriptome data collected in the field [6, 8, 9, 16]. We previously developed a statistical model that predicts transcriptome dynamics of rice leaves in the field from meteorological data [6, 8, 16]. The model suggested that transcriptome dynamics are predominantly governed by endogenous diel rhythms, ambient temperature, solar radiation, and plant age. The model well predicted the transcriptome dynamics of rice leaves grown in different years from the data used to develop the model. However, the model has low prediction accuracy at rare conditions in the field, such as extraordinarily high temperatures. This is due to the narrow range of environmental parameters and their correlation in the field (e.g., high temperature during the day and low temperature during the night).

One solution to this problem is adding transcriptome data from controlled conditions with a wide range of environmental parameters while ensuring that the parameters are not correlated. The model trained with field data incorporates the rice plant’s responses to typical and fluctuating environmental conditions and developmental stages commonly observed in the field. Therefore, incorporating transcriptome data collected under controlled conditions can significantly enhance the model, even if these conditions lack environmental fluctuations and differ in developmental stages compared to field-grown rice. However, since transcriptome data under typical field conditions can already be obtained directly from field-grown plants, reproducing such conditions in controlled environments is unlikely to improve the model. In contrast, collecting and incorporating transcriptome data from rice grown under atypical conditions unlikely to occur in natural field environments can significantly improve the model. Nevertheless, setting up many growth chambers is logistically difficult [19].

In this study, we developed cost- and space-effective GCs. Using the GCs, we conducted a massive transcriptome analysis of rice leaves grown under 73 conditions: three photoperiods, five temperature levels in the light, and five temperature levels in the dark. Using transcriptome data of the GC-grown rice and field-grown rice as input data, we extended our statistical model for predicting gene expression from meteorological data, highlighting the importance of irradiance in transcriptome regulation, a contrary conclusion from the previous study [8]. Our results contribute to a deeper understanding of plant responses to the environment in the natural environment and the agricultural field.

## Results

### Systematic measurement of rice transcriptome dynamics across 73 growth chamber conditions

**Fig. 1:**
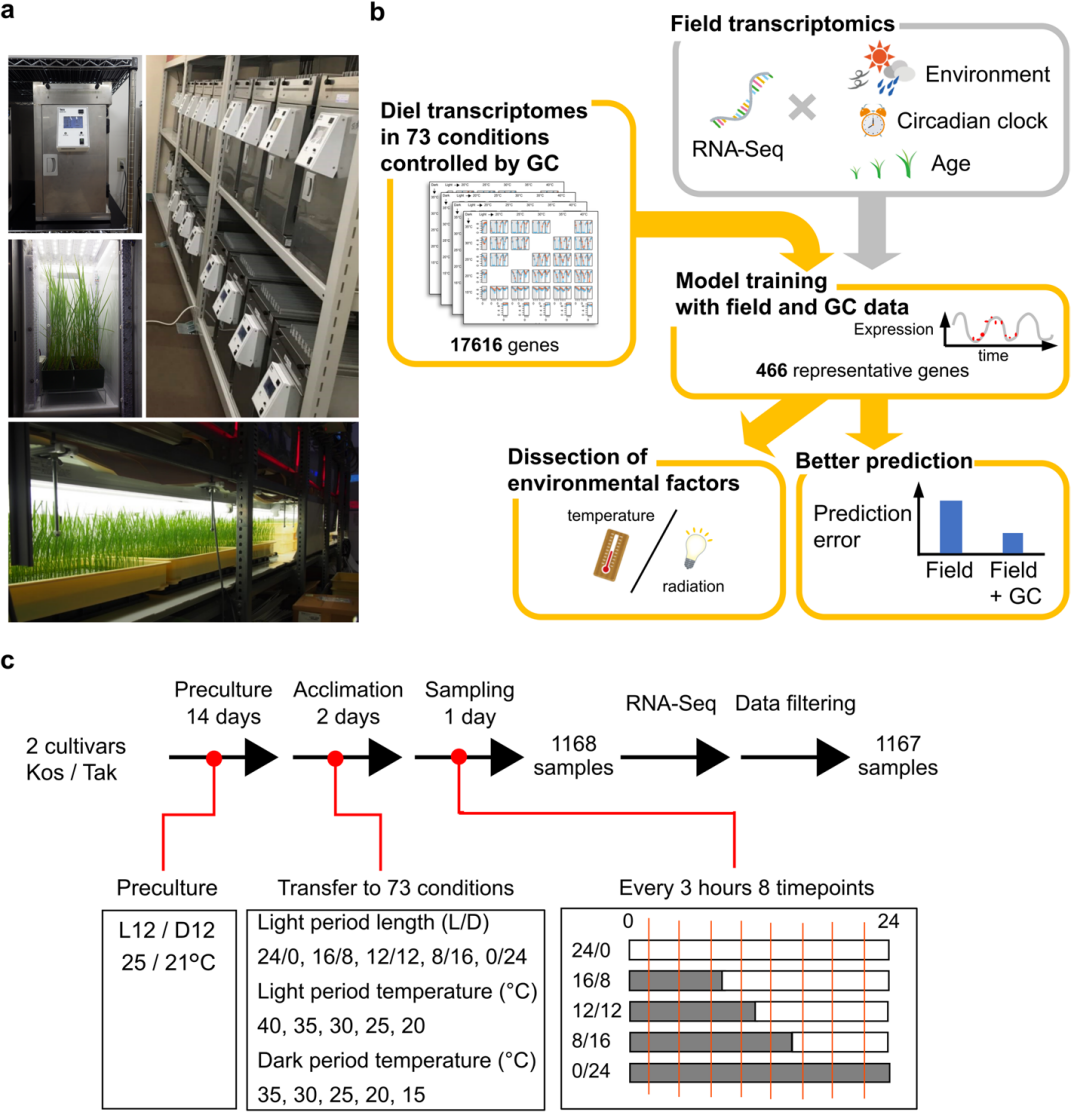
Systematic approach analyzing rice transcriptome dynamics across 73 growth chamber conditions for plant environmental response insights. **a** GC and the preculture condition. Front view of GC (upper left), rice grown in GC (middle left), the parallel control of GC (right), and the preculture condition (bottom). **b** Schematic diagram of this study. In our previous field transcriptomics study, we developed a statistical model to predict the transcriptome dynamics of rice leaves in the field. The main factors considered in the model included circadian clock, temperature, solar radiation, and plant age. In this study, we obtained diel transcriptome data from the 73 controlled conditions where irradiance and temperature were independently varied. Training the model with the transcriptome data from controlled and field conditions allowed us to dissect causal environmental factors and more accurately predict transcriptome dynamics in the field. **c** Experimental design of sampling and transcriptome analysis across 73 growth chamber conditions

**Fig. 2:**
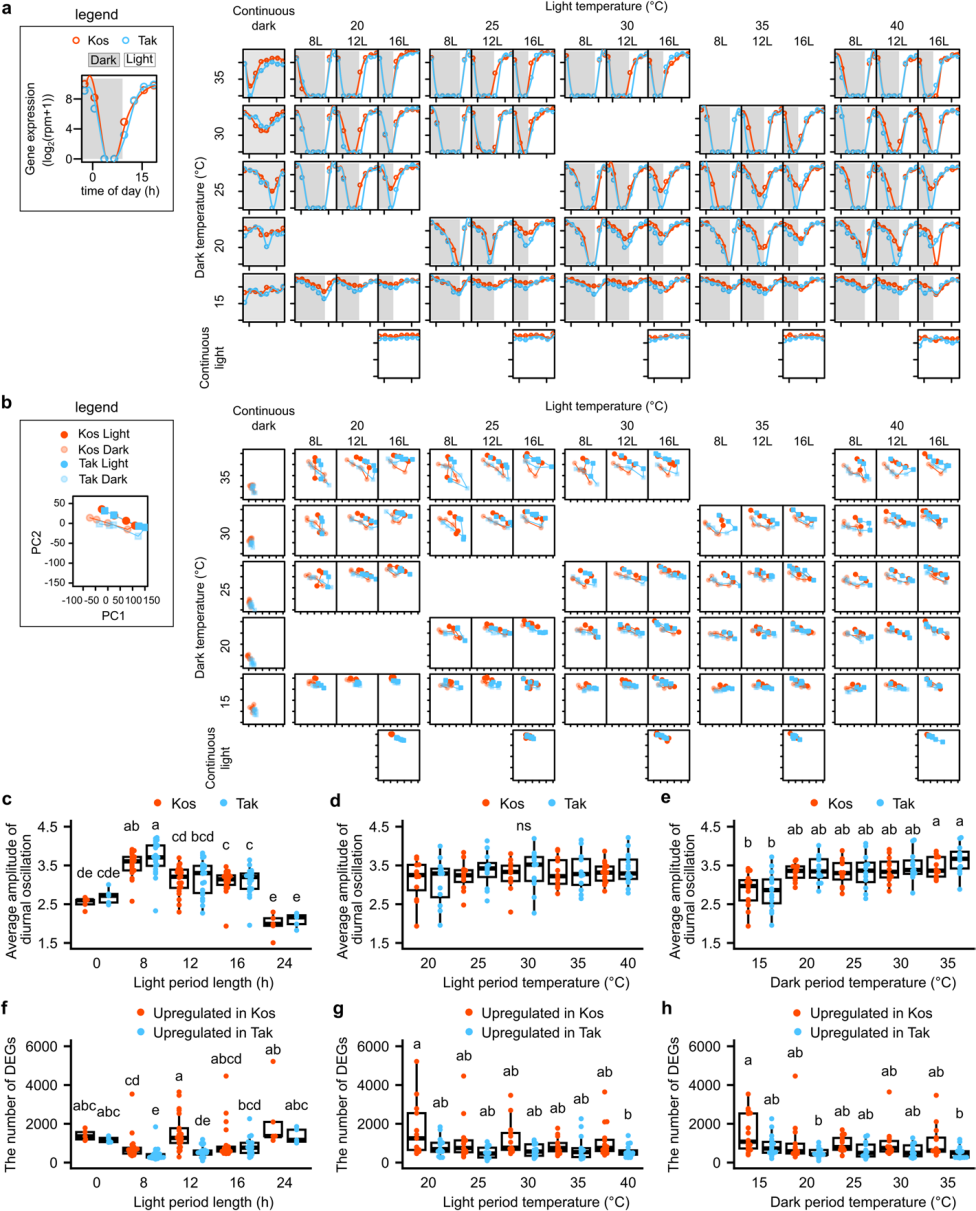
Systematic measurement of rice transcriptome dynamics across 73 growth chamber conditions. **a** Expression levels of *OsGI* (*Os01g0182600*) in 73 conditions. In each subpanel, expression levels (log_2_(rpm + 1)) of Koshihikari and Takanari at each condition are shown in red and blue, respectively. White and gray zones indicate light and dark periods, respectively. The subpanels are arranged according to the light/dark temperature. Within each group, the 8L/16D, 12L/12D, and 16L/8D photoperiod conditions are arranged from left to right, whereas the 0L/24D and 24L/0D conditions are displayed separately. The x-axis, y-axis, and their scale values in the subpanels are the same as those in the legend. **b** Principal component analysis of transcriptome in 73 conditions. In each subpanel, PCA of Koshihikari and Takanari at each condition are shown in red and blue, respectively. Solid and transparent dots indicate samples at light and dark conditions, respectively. For each cultivar, the dots are connected by lines in the order of sampling time. The subpanels are arranged in the same order as in **a**. The x-axis, y-axis, and their scale values in the subpanels are the same as those in the legend. Genome-wide average amplitudes of diel oscillation of gene expressions at each condition, which is classified by **c** light period length, **d** light period temperature, and **e** dark period temperature. The number of differentially expressed genes (DEGs) between Koshihikari and Takanari at each condition, which is classified by **f** light period length, **g** light period temperature, and **h** dark period temperature. In **c–h**, conditions that share the same letter are not significantly different from each other, as determined by multiple comparisons using the Steel–Dwass test (*p* ≥ 0.05)

We conducted a massive transcriptome analysis of rice leaves grown in 73 conditions, combining photoperiod length and temperatures in light and dark periods (Fig. 1 and Additional file 2: Table S1). We developed a cost- and space-effective growth chamber (GC), enabling multi-environment tests in controlled conditions (Fig. 1a). By utilizing the GC, we grew two cultivars of rice (Koshihikari and Takanari), both of which were used for transcriptome analysis to construct our previous model [6] (Fig. 1b, c). Koshihikari is a leading japonica cultivar in Japan, while Takanari is a high-yield indica cultivar with some indica-specific features, such as a high photosynthetic rate [30]. Both cultivars are typically grown in the field under a photoperiod of approximately 12 to 14 h and temperatures ranging from 15 to 35 °C in Japan. Before the experiment, we grew all the rice plants for 14 days at 25/21 °C under 12L/12D photoperiod (Fig. 1c). Subsequently, we transferred the plants to the GCs with different conditions, where they were left to acclimate for 2 days. The leaves were sampled on the third day. Although having replicates at the time points would have been ideal, the number of samples was limited due to space limitations in the GCs. Given this limitation, we prioritized increasing the number of time points over biological replication, as this provides more valuable information and is more beneficial for model training. Therefore, in each condition, we sampled rice leaves from one plant per cultivar every 3 h for 8 times (1168 total samples: 8 time points × 2 cultivars × 73 conditions) and conducted RNA-Seq analysis (Additional file 2: Table S2). During the RNA-Seq analysis, replacement of the labeling of samples occurred. By predicting air temperature at the sampling time point from transcriptome data, we corrected the labeling of samples (Additional file 1: Figs. S1 and S2). After omitting samples with different genotypes from the labeling of the sample (Additional file 1: Fig. S1), 1167 samples were used for further analysis (Figs. 1c and 2a). By incorporating the transcriptome data across 73 conditions into the model, we aimed to dissect the environmental factors affecting rice transcriptome dynamics and to improve the prediction of transcriptome dynamics from meteorological data (Fig. 1b).

### Independent effects of light period length and temperature on rice gene expression and cultivar-specific responses

Our massive transcriptome data under controlled conditions allow us to disentangle the independent effect of light period length and temperature on rice gene expression. To summarize the diel transcriptome dynamics, we conducted principal component analysis (PCA) (Fig. 2b). Transcriptomes in continuous dark conditions (0L/24D) were separated from the other conditions. Diel changes in the transcriptome became pronounced under short-day conditions (8L/16D) and with increasing dark temperatures. We also evaluated transcriptome similarity between conditions using t-SNE, another method of summarizing transcriptome data (Additional file 1: Fig. S3). Transcriptomes in continuous dark conditions (0L/24D) were separated from the other conditions, which is consistent with the results of PCA (Fig. 2b). The other samples were mainly classified by cultivar and light/dark (Additional file 1: Fig. S3a). Samples at similar time points (Additional file 1: Fig. S3b) and temperature (Additional file 1: Fig. S3c) were clustered closely. To evaluate the diel changes in the transcriptome, we calculated the amplitude of diel oscillation of gene expression (Fig. 2c–e and Additional file 1: Fig. S4). Under the typical conditions for Koshihikari and Takanari in the field (e.g., 25/20 °C, 30/20 °C, and 30/25 °C [light/dark temperatures] under 12L/12D conditions), the amplitudes (log_2_(rpm + 1)) of diel oscillation were 3.37, 3.48, 3.55 for Koshihikari and 3.49, 3.70, 3.53 for Takanari, respectively. Under 73 conditions, the amplitudes of diel oscillations varied more widely. The amplitude of diel oscillation tended to be lower under continuous dark (0L/24D) and continuous light (24L/0D) conditions, 2.60 and 2.72 for Koshihikari and Takanari under 0L/24D, and 1.99 and 2.16 under 24L/0D, respectively, compared with the other conditions (Fig. 2c). On the other hand, the amplitude of diel oscillation was significantly higher in 8L/16D (with median values of 3.61 and 3.71 for Koshihikari and Takanari, respectively) than in 12L/12D (3.21 and 3.30) and 16L/8D (3.14 and 3.20) for both cultivars; the amplitude was higher in short-day conditions. The amplitude of diel oscillation was neither different between cultivars nor affected by light period temperature (Fig. 2d). However, it was affected by dark period temperature, where the amplitude of diel oscillation tended to be lower at 15 °C (with median values of 2.80 and 2.94 for Koshihikari and Takanari, respectively) than in the other conditions and significantly lower than that at 35 °C (3.34 and 3.64) (Fig. 2e). This is consistent with the previous study, which showed that the amplitude of diel oscillation of gene expression was lower in winter than in summer in *Arabidopsis halleri* subsp. *gemmifera* [7]. Further investigation is required to clarify whether 15 °C during the daytime also decreases the amplitude of the diel oscillation of gene expression.

To clarify the difference in the environmental response of Koshihikari and Takanari, we compared the number of differentially expressed genes (DEGs) between the two cultivars at each condition. Because Koshihikari and Takanari belong to different subspecies (japonica and indica, respectively), some genes showed cultivar-specific gene expression [6, 31–33]. To elucidate the effect of environmental conditions, we excluded cultivar-specific genes from the DEGs (Fig. 2f–h). Cultivar-specific genes were defined as those where the between-cultivar difference in the average expression (log_2_(rpm + 1)) calculated across the 73 conditions was greater than 2, and the average expression in the cultivar with lower expression was less than 0.5. The number of cultivar-specific genes in Koshihikari and Takanari was 483 and 110, respectively. Under typical conditions for Koshihikari and Takanari in the field (25/20 °C, 30/20 °C, and 30/25 °C [light/dark temperatures] under 12L/12D), the number of DEGs was relatively low. The number of DEGs with higher expressions in Koshihikari was 291, 570, and 1048, while those with higher expressions in Takanari were 94, 206, and 350, respectively. The number of DEGs tended to be higher in 0L/24D (with median values of 1350 and 1195 for Koshihikari and Takanari, respectively) and 24L/0D (1364 and 1166) than in the other conditions (Fig. 2f). The absence of environmental fluctuations under constant conditions may exaggerate cultivar differences. The number of DEGs with higher expression in Koshihikari was significantly higher than that with higher expression in Takanari in 8L/16D (with median values of 657 and 315 for Koshihikari and Takanari, respectively) and 12L/12D (1269 and 508) conditions, but the difference between the cultivars was not seen in the other conditions (Fig. 2f). Although the number of DEGs higher in Koshihikari or Takanari was not significantly different at any light or dark temperatures (Fig. 2g, h), the number of DEGs tended to be higher in Koshihikari than in Takanari in the low-temperature conditions: 20 °C in the light period and 15 °C in the dark period. This difference might result from the cold tolerance of the japonica cultivar compared with indica cultivars [34]. Collectively, our results demonstrated that diel transcriptome dynamics and their cultivar differences were more pronounced under atypical field conditions. We also highlighted the effect of light period length on the amplitude of gene expression and the different responses to a low temperature between Koshihikari and Takanari.

### Co-expression gene network analysis identifies genes responsive to temperature

**Fig. 3:**
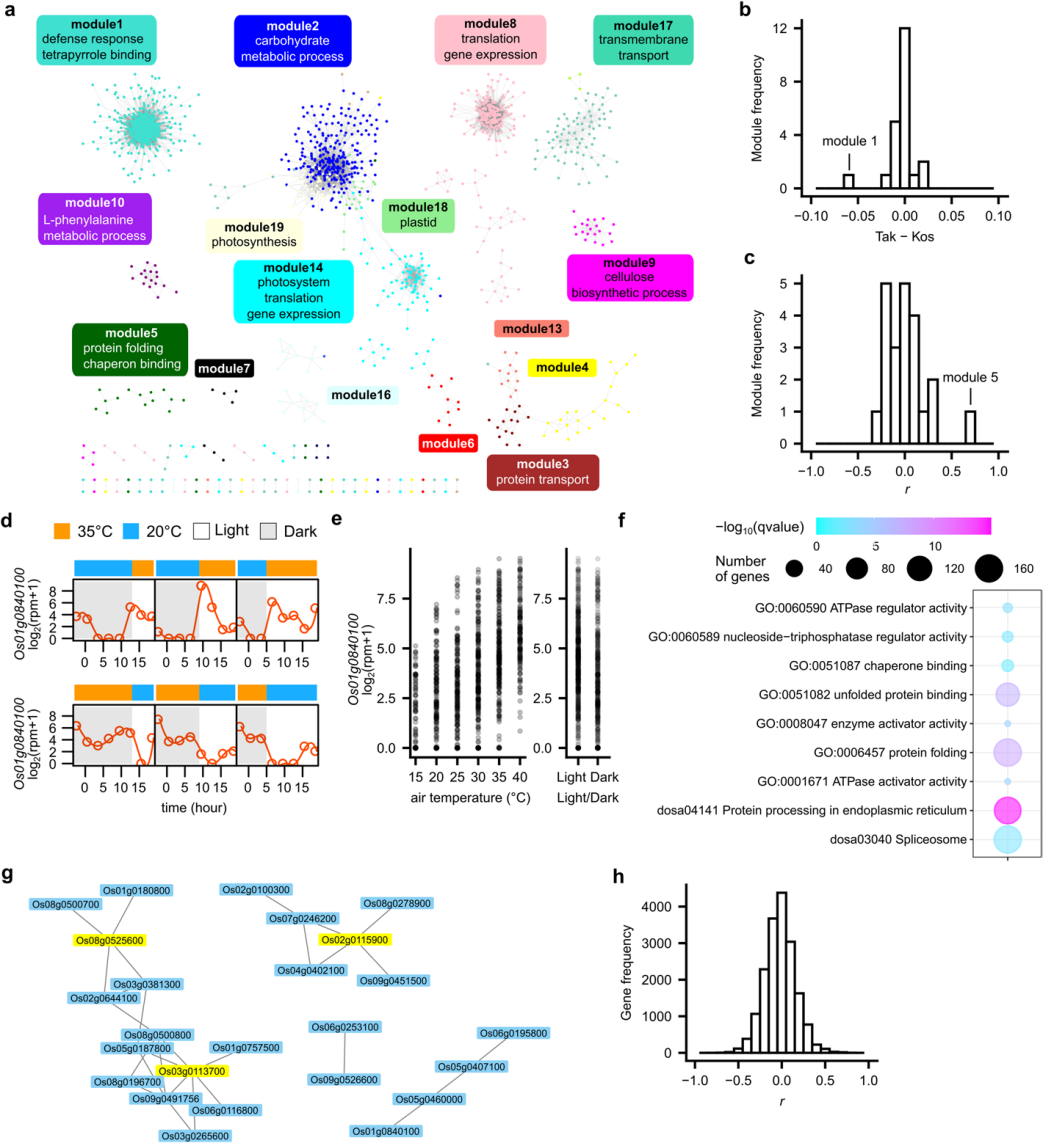
Identification of genes responsive to environmental conditions. **a** Co-expression network constructed by WGCNA. Representative gene ontology (GO) enriched in each module is shown. **b** A histogram showing the differences in mean expression of module eigengenes of Koshihikari and Takanari. **c** A histogram showing Pearson’s correlation coefficient between the expression of module eigengenes and air temperature. Expression levels of a gene encoding *Hsp 70* (*Os01g0840100*) in module 5 **d** at 35/20 °C (L/D) and 20/35 °C (L/D) conditions in Koshihikari and **e** 73 conditions in Koshihikari and Takanari at each air temperature or light/dark condition. **f** Gene ontology and KEGG pathway significantly enriched in the temperature-responsive module 5. **g** Gene network and hub genes of the temperature-responsive module 5. **h** A histogram showing Pearson’s correlation coefficient between the expression of each gene and air temperature

We analyzed the co-expression gene network using WGCNA [35]. The co-expression gene network was composed of 22 modules (clusters of co-expressed genes) with the number of genes per module ranging from 20 to 1416 (Fig. 3a). To find modules that have different expression patterns between Koshihikari and Takanari, we compared the mean value of eigengenes between the two cultivars, which was defined as the first principal component of the genes within a module (Fig. 3b). Module 1 was preferentially expressed in Koshihikari. In module 1, genes annotated for defense response (GO:0006952) and tetrapyrrole binding (GO:0046906) were significantly enriched (Fig. 3a and Additional file 2: Table S3). This result is consistent with the previous studies showing the preferential expression of genes related to disease resistance [32, 33] and porphyrin and chlorophyll metabolism [33] in japonica rice, and the differences in blast resistance genes between Koshihikari and Takanari [36].

To detect specifically temperature-responsive modules, we calculated Pearson’s correlation coefficient (*r*) between temperature and the expression level of the eigengenes (Fig. 3c). Module 5 showed a strong positive correlation with temperature (*r* = 0.69), suggesting that module 5 is composed of temperature-responsive genes (Fig. 3c–e and Additional file 1: Figs. S5 and S6). On the other hand, the other modules showed a low correlation with temperature. The expression of the genes belonging to the modules tended to be constant (e.g., module 2) or regulated by the light/dark cycle (e.g., modules 4, 8, 17, and 18) (Additional file 1: Fig. S5).

To characterize the modules, we tested the enrichment of genes with gene ontology (GO) annotation and KEGG pathway in the modules. Genes annotated for chaperone binding (GO:0051087), protein folding (GO:0006457), and protein processing in the endoplasmic reticulum (KEGG pathway: dosa04141) were significantly enriched in module 5 (Fig. 3a, f and Additional file 2: Tables S3 and S4), indicating that genes belonging to module 5 is composed of heat stress-related genes (Additional file 2: Table S5). We identified three hub genes in module 5 that can have a central role in the regulation of the module (Fig. 3g). *Os02g0115900* encodes binding immunoglobulin protein (BiP), which is an endoplasmic reticulum-localized Hsp70 chaperone [37]. *Os03g0113700* encodes mitochondrial Hsp70 [38]. *Os08g0525600* encodes FK506 binding protein, which is involved in stress response [39]. We also calculated the Pearson’s correlation coefficient (*r*) of gene expression and temperature to extract genes responsive to air temperature. *r* ranged from − 0.76 to 0.77 (Fig. 3h and Additional file 2: Tables S6 and S7). Among the 91 genes with positive correlation (*r* > 0.5), 38 genes were members of module 5, and these genes include heat shock protein genes (Additional file 2: Tables S5 and S6). Expressions of 60 genes were negatively correlated with air temperature (*r* <  − 0.5) (Additional file 2: Table S7). Among the 60 genes with negative correlation (*r* <  − 0.5), *Os09g0551600* showed the lowest *r*. *Os09g0551600* encodes an HMG protein (nucleosome/chromatin assembly factor D protein). The HMG protein is involved in various DNA-dependent processes, including transcription, recombination, and DNA repair [40]. *Os05g0537400* showed the second lowest *r*. *Os05g0537400* encodes protein phosphatase *PP2C50*, a major negative regulator of ABA signaling regarding stomata closing in rice [41]. These genes might be important for the adjustment to air temperature change in rice. Collectively, our results identified the temperature-responsive genes and the difference in gene expression between rice cultivars. Our approach, which uses diel transcriptome data across a comprehensive range of temperature conditions, allows for a more accurate evaluation of temperature effects by minimizing the influence of confounding factors such as time-of-day variation, compared to analyses based on single time point measurements. Further analysis of temperature-responsive genes will contribute to plant response to temperature.

### Radiation is a better predictor of expression than temperature for the majority of genes

**Fig. 4:**
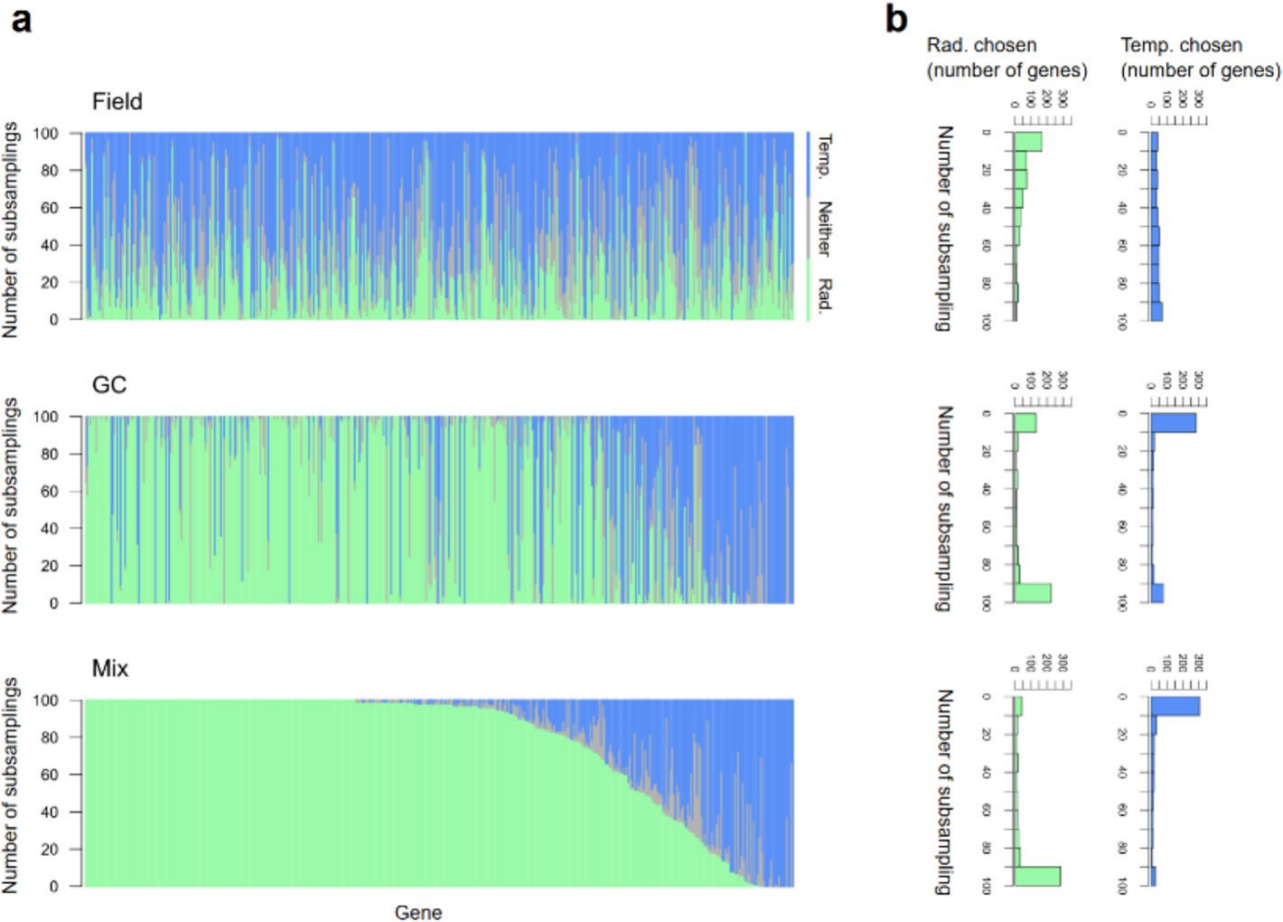
The number of times temperature or radiation (or neither) was chosen as the predictor of gene expression (Koshihikari). **a** A single vertical line represents a gene whose green, blue, and gray parts show the frequency where radiation, temperature, or neither was chosen (among the 100 data subsampling). The 466 genes are sorted according to the frequency at which radiation was chosen in the model training with mixed (GC + field) data. **b** Histograms showing how frequently temperature or radiation was chosen

We then compared radiation and temperature as the predictors of gene expression dynamics (Fig. 1b). Under the field conditions, radiation and temperature are correlated with each other. Here we are interested in how the data from the GC, where radiation and temperature were systematically varied independently, enabled the choice of the better predictor. We thus trained statistical models for gene expression dynamics using different training data sets: GC data, field data, or a mixture of them (50% GC and 50% field). Field data were obtained over 3 years from rice plants grown under typical conditions for Koshihikari and Takanari in Japan (see Methods for details). We mainly report the results for Koshihikari in the main text, but the results for Takanari were qualitatively the same (Additional file 1: Figs. S7 and S8). We focused on 474 genes representing various expression patterns in the field. These genes were chosen based on the clustering of microarray data analyzed by Nagano et al. [8] and were representative of each cluster of genes with similar expression (see Methods for details). Among the 474 representative genes, 8 genes showed too low expression levels for model training. We thus trained models for the remaining 466 genes (one model for a gene). We used the R package FIT [16] for the model training. In the model of FIT, we consider plant’s age (days after planting), circadian clock (time of day), and environmental responses as the predictors of gene expression. The sub-model of environmental response consisted of the sum of a gate function (diel changes in environmental responsiveness) applied to environmental variables over a period of time in the past. Radiation or temperature, the two environmental variables most influential on gene expression [8], was chosen for each gene. In the present study, we did not consider the model to consist of both temperature and radiation due to constraints on computation time and parameter optimization (see Methods for details). Because we used different training data sets (GC, field, and the mixture of them), we randomly subsampled 512 data points from each set to standardize the size of different training data. We repeated the subsampling and model training 100 times. We can thus calculate how frequently radiation or temperature was chosen as the predictor for each gene (Fig. 4). When trained with field data, temperature was more frequently chosen than radiation for the majority of the genes (338 out of 466 genes with successful model training), corroborating a previous result with field data [8]. However, when the models were trained with GC or mixed data, radiation was more frequently used than temperature as the predictor of the majority of the genes (GC: 308/466 genes; mix: 378/466 genes) (Additional file 2: Table S8). The choice of the predictor was also more consistent across data subsampling when we used GC or mixed data than field data (Fig. 4b). To characterize the genes, we tested gene ontology (GO) annotation and KEGG pathway enrichment in the genes belonging to clusters whose representative genes were the 338, 308, or 378 genes. While no GO terms and KEGG pathways were significantly enriched in the genes belonging to the clusters of 308 genes, genes annotated for antenna proteins of photosynthesis (dosa00196) and the spliceosome (dosa03040) were significantly enriched in the clusters of 338 genes (Additional file 2: Table S9). Additionally, genes annotated for protein transport and localization (e.g., GO: 0015031 and GO: 0008104) were significantly enriched in the clusters of 378 genes (Additional file 2: Table S9). We also conducted GO and KEGG enrichment analyses on the gene sets better predicted by the other predictor (128, 155, and 85 genes in the field, the GC, and the mixed models, respectively). No significant GO terms and KEGG pathway were significantly enriched in the clusters of 128 genes, and only the ribosome pathway (dosa03010) showed significant enrichment in the clusters of 155 and 85 genes (Additional file 2: Table S9). Similar results were obtained for Takanari (Additional file 2: Tables S10 and S11).

To identify the genes for which the input data influenced the choice of predictors, we extracted the representative genes for which temperature was consistently chosen with field-data training and radiation was consistently chosen with mixed data training (criterion for consistency: ≥ 80% subsampling). The number of the representative genes extracted was 92 for Koshihikari and 101 for Takanari, where 38 genes were shared (Additional file 2: Table S12). Because individual genes represent clusters of different sizes (number of genes), we estimated the genome-wide impact. Among the 15,907 genes belonging to the 466 clusters, 2929 (18.4% of the total genes) were identified for Koshihikari and 3208 genes (20.2%) for Takanari, with 1167 genes (7.3%) shared. To characterize the genes, we tested the enrichment of genes with gene ontology (GO) annotation and KEGG pathway. While genes significantly enriched in Koshihikari were not observed, genes annotated for vesicle-mediated transport (e.g., GO: 0016192), 1,3-beta-D-glucan biosynthesis (e.g., GO: 0003843), and proteasome (e.g., dosa03050) were significantly enriched in Takanari (Additional file 2: Table S13).

We further analyzed genes for which the same predictor (radiation or temperature) was consistently chosen in both field and mixed data training. For radiation, we extracted 18 and 11 representative genes in Koshihikari and Takanari, respectively, with 4 shared (Additional file 2: Table S14), corresponding to 526 (3.3%) and 251 (1.6%) genes genome-wide, with 93 shared genes (0.6%). These were significantly enriched for flavonoid biosynthesis (e.g., dosa00941) in both cultivars, and for lipid transport (e.g., GO:0006869) in Takanari (Additional file 2: Table S15). For temperature, 4 and 19 representative genes were identified for Koshihikari and Takanari, respectively, with 1 shared (Additional file 2: Table S16), corresponding to 96 (0.6%) and 687 (4.3%) genes genome-wide, with 14 shared genes (0.1%). Enrichment analysis revealed significant associations with protein processing (e.g., dosa04141) in both cultivars and with protein biosynthesis/metabolism (e.g., GO:0043043, dosa03010) in Takanari (Additional file 2: Table S17).

### Mixed data improves the prediction of gene expressions in the field

**Fig. 5:**
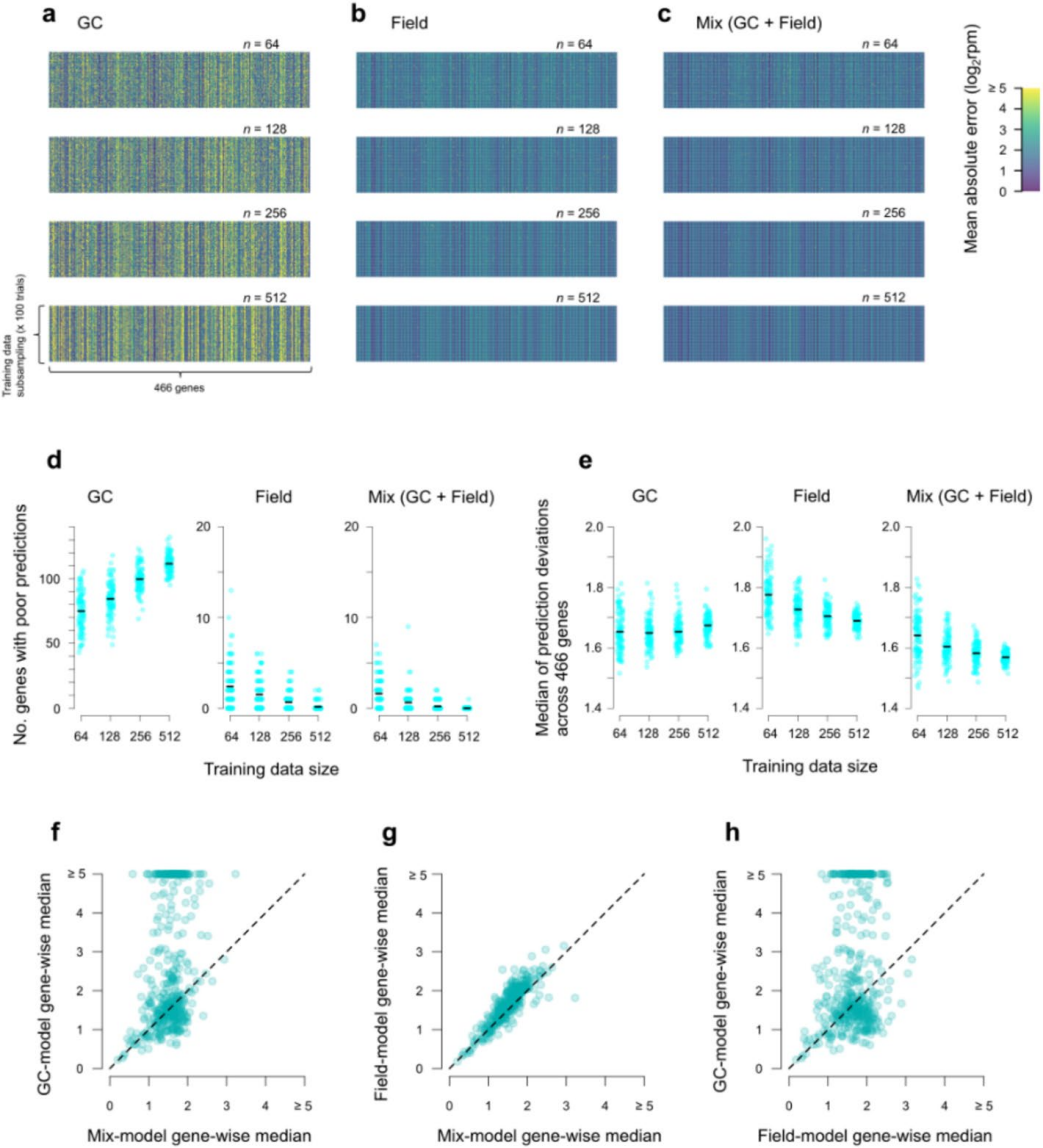
Prediction performances of gene expression models trained with different data sets in Koshihikari. The performances were evaluated by applying the models to the field test data (not included in the training data). **a**–**c** Heat maps of mean absolute errors (MAE). The GC + field (**c**) models were trained with a mixture of GC and field data (50% each). Training data size (*n*) was also varied. **d–h** show summary statistics of **a–c**. **d** The numbers of genes with poor predictions (log_2_rpm ≥ 5). **e** Medians (across 466 genes) of the MAE values. In **d** and **e**, data points correspond to training data subsampling, and black horizontal lines represent means. **f–h** Gene-wise medians of the MAE values compared between models trained with different data sets

To examine whether model predictions improve quantitatively due to the use of GC results in model training, we evaluated the models’ performances by applying the models to field-grown plants (*n* = 615) that were not included in the training data. Because the results can depend on training data sizes, we also varied the sizes of subsampled training data (*n* = 64, 128, 256, and 512; subsampled 100 times for each size). We calculated mean absolute error (MAE) to measure the model performance by applying the trained models to the test data. Thus, we obtained 100 MAE values for 466 genes (Fig. 5a–c). Prediction performance varied substantially across genes, and these differences were relatively consistent across training data subsampling.

As a general trend, the use of mixed data (GC + field) resulted in better predictions than other data sets (Fig. 5d). We considered MAE ≥ 5 (in log_2_rpm) as poor predictions. The number of genes with poor predictions almost always exceeded 50 when we used GC data alone for model training (Fig. 5d). The genes with poor predictions were much fewer with field-alone or mixed data. The number of genes with poor prediction decreased with increasing training data size for field and mixed training data. Notably, the largest mixed data resulted in the virtual absence of genes with poor predictions. Larger training data sizes for GC models decreased model performances, suggesting overfitting. Medians of the MAEs calculated across genes, which reflect the average performances of the models trained with different data sets, were small when the models were trained with large mixed data (Fig. 5e). On average, MAE median was smaller for GC than for field models (Fig. 5e). Results in Fig. 5d and e suggest that in GC models, whereas many genes showed poor predictions, other genes showed good prediction performance; for some genes GC data appear more informative than field data to predict the field test data.

To identify such genes, we then summarized the MAE in Fig. 5a–c gene-wisely (rather than subsampling-wisely) by calculating medians for the largest training data of *n* = 512 (i.e., average performance of models for a particular gene) (Fig. 5f–h). When the mixed model performances are plotted against field model performances, 360 out of the 466 genes showed better predictive performance with the mixed model than with the field model, as indicated by points above the diagonal line in Fig. 5g (Additional file 2: Table S18). Similarly, when the GC model performances are plotted against field model performances, 202 out of the 466 genes showed better predictive performance with the GC model than with the field model, as indicated by points below the diagonal line in Fig. 5h (Additional file 2: Table S18). Of these 202 genes, 192 overlapped with the 360 genes that showed better performance in the mixed model than in the field model. As a result, similar GO terms or KEGG pathways were significantly enriched in the genes belonging to clusters of 202 genes (Additional file 2: Table S19). In particular, genes related to protein synthesis and metabolism, such as ribosome (GO: 0005840) and peptide metabolic process (GO: 0006518), as well as intracellular components, such as intracellular anatomical structure (GO: 0005622), were significantly enriched in the genes belonging to clusters of both the 360 genes and 202 genes (Additional file 2: Table S19). Similar results were obtained for Takanari (Additional file 1: Fig. S8; Additional file 2: Tables S20 and S21). These results suggest that genes better predicted by the GC model also contributed to the improved performance of the mixed model.

In the GC data, we created conditions with both positive and negative temperature-irradiance correlations (Figs. 1c and 2a), yet whether we used only positively or negatively correlated data (or both) did not affect the outcome (Additional data 1: Fig. S9).

## Discussion

The correlation of irradiance and temperature in the field makes it difficult to evaluate the irradiance and temperature response of plants. In this study, by combining massive transcriptome analysis of rice leaves grown under controlled conditions and statistical modeling predicting rice transcriptome from meteorological data, we successfully distinguished the effect of irradiance and temperature on the plant transcriptome.

When we trained the models with field data, many gene-specific models chose temperature and radiation at intermediate frequencies (~ 50), suggesting that the strong correlation between temperature and radiation in the field made it difficult to identify the environmental variable affecting the gene expression levels (Fig. 4). On the other hand, models trained with either the GC data or mixed data were more consistent in the choice of the environmental variables (~ 0 or ~ 100). These results suggest that using laboratory (i.e., GC) data where temperature and radiation do not correlate enables the models to choose the appropriate environmental variable to predict gene expression. However, model training only with the GC data led to poor predictions on field data, with the evidence of overfitting on GC data with increasing training data size (Fig. 5). The GC data included only very young plants. In contrast, the field test data included plants of various ages. The models trained with the GC data thus extrapolated for plant age to make predictions. Other factors such as absence of fluctuations of the environmental factors in GC might also be involved in the poor predictions of the GC model on field data. Field and GC data appear complementary to each other. GC data enabled the choice of better predictors (Fig. 4), but GC data alone led to poor parameter estimation (Fig. 5). The mixture of GC and field data has enabled the choice of better predictors and good parameter estimation, which explains the high prediction performance of the models trained with the mixed data. The test data we used were collected in a relatively similar environment to that of the field data for training. The performance differences between models trained with different data sets may become more pronounced when tested using data from novel environments (e.g., higher latitude).

Our results highlight the importance of irradiance in the transcriptome regulation. Previous studies using statistical modeling [8, 9] have suggested that temperature was more important than irradiance. A transcriptome study showed that the diel change of expression of *Brachypodium distachyon* was mainly affected by temperature [42]. In addition, an increase in the night temperature was found to have affected the diel change of transcriptome by regulation of PRR genes of rice panicles [43]. However, the temperature in these studies was confounded with irradiance, although continuous light/dark or temperature conditions exist in MacKinnon et al. [42]. This might have apparently increased the relative importance of temperature for transcriptome regulation in the previous studies.

Expression of some genes is known to be affected by both light and temperature [18, 42, 44]. On the other hand, our statistical model chose only one environmental parameter (irradiance, temperature, or none) and cannot consider the joint regulation by both irradiance and temperature (see Methods for details). Although it is possible to consider a model with multiple environmental factors, it would become more complex. The statistical model in Matsuzaki et al. [9] considers both irradiance and temperature, but only 25 genes associated with the circadian clock were analyzed due to the high calculation cost. Using the model, Matsuzaki et al. [9] showed that some circadian genes were affected by both irradiance and temperature, but more genes were affected by temperature than irradiance. This might be because field transcriptome and meteorological data, where irradiance and temperature are correlated, were used for the model training.

Using GC, we produced data where temperature and irradiance are independently varied, which appear to have improved the prediction of our model with mixed training data. In particular, we made the conditions where the temperature in the dark was higher than that in the light (Fig. 1), sometimes called negative DIF (day-night temperature difference). Negative DIF conditions have been used to research plant circadian clock and phytohormone signaling [45, 46]. In addition, negative DIF is also used to control the growth of horticultural crops [47]. However, few studies have evaluated the effect of the condition on plants utilizing the omics approach. Counterintuitively, negative DIF was not especially informative for model training (Additional file 1: Fig. S9). Future studies will clarify the optimum experimental designs to improve the prediction of the model effectively.

## Conclusions

In this study, we extended our statistical modeling for predicting gene expression from meteorological data in the field by incorporating rice transcriptome systematically measured under 73 controlled conditions. Our study revealed that rice transcriptome dynamics in the field are primarily regulated by irradiance. Furthermore, integrating both field and controlled condition data substantially improved the precision and accuracy of the model’s predictions. Since the transcriptome data can be used for trait prediction [48–53], improvement of the model used in this study will contribute to improving the accuracy of the trait prediction. Trait prediction is essential for the evaluation of the effect of climate change on crop production [54]. In this case, adding data obtained from extreme conditions (e.g., high or low temperature) will be effective. Improving trait prediction through the statistical model will contribute to understanding plant environmental response in the field and assessing climate change on plants.

## Methods

### Plant materials and growth conditions

In this study, we developed a high irradiance growth chamber (GC, ECP101, TECS Inc., Ibaraki, Japan) that can be controlled in parallel by one PC (Fig. 1a). The GC can control irradiance level and air temperature. The output value of each LED can be set from 0 to 100%. In this study, we set the output value to 100%. The irradiance level was 1300 and 350 µmol photon m^−2^ s^−1^ (photosynthetic photon flux density [PPFD]) at the top (5 cm from the light source) and the bottom of the GC, respectively (Fig. 1a). The temperature was set to 15–40 °C. The GC can record temperature and relative humidity every minute.

A japonica rice (*Oryza sativa* L.) cultivar, “Koshihikari,” and an indica rice cultivar, “Takanari,” were used in this study. Seeds were sterilized in a 2.5% (v/v) sodium hypochlorite solution for 30 min and then soaked in water at 25 °C for 4 days. Germinated seeds were sown in a cell tray filled with nursery soil (N:P_2_O_5_:K_2_O = 0.6:1.2:1.0 g/kg). In the preculture, plants were grown for 14 days at 25/21 °C and relative humidity of 55 ± 10/70 ± 10% under a 12-h light/12-h darkness (12L/12D) photoperiod. Fluorescent lamps were used for the light source (FHF32EX-N–H, Iwasaki Electric Co., Ltd., Tokyo, Japan). The irradiance level was 400 ± 20 µmol photon m^−2^ s^−1^ (PPFD) at 5 cm from the light source (Fig. 1a). Plants were grown while maintaining plant height so that plant tips were 5 cm from the light source (Fig. 1a). Spot coolers were used to avoid burnt leaves due to the heat from the light source. Plants were then transferred to GC set to each condition (Fig. 1c). Environmental conditions in GC were composed of a combination of light period length (0L/24D, 8L/12D, 12L/12D, 16L/8D, and 24L/0D), light period temperature (20, 25, 30, 35, and 40 °C), and dark period temperature (15, 20, 25, 30, and 35 °C). Conditions with the same light and dark period temperatures were excluded. As a result, experiments with 73 conditions were conducted (Fig. 1c and Additional file 2: Table S1). Experiments in 73 conditions were divided into 4 terms. In each term, sampling was conducted in 16, 23, 21, and 13 conditions (Additional file 2: Table S1). After transferring the plants to each condition, they were acclimatized for 48 h. Sampling was then conducted every 3 h for 24 h (1.5, 4.5, 7.5, 10.5, 13.5, 16.5, and 19.5 h after the acclimation period, 8 times in total) (Fig. 1c). Under each condition, the uppermost, fully expanded leaves were sampled from one plant per sampling point, frozen in liquid nitrogen, and stored at − 80 °C for future use. At each sampling time point, sampling was completed within 20 min.

### RNA-Seq analysis

Frozen samples were homogenized with TissueLyser II (Qiagen, Hilden, Germany), and total RNA was extracted using the Maxwell 16 LEV Plant RNA Kit (Promega, Madison, WI, USA). RNA concentration was measured using the broad-range Quant-iT RNA Assay Kit (Thermo Fisher Scientific, Waltham, MA, USA). Total RNA (500 ng) was used as the input of each sample for library preparation. Library preparation for RNA sequencing was conducted using Lasy-Seq [55] version 1.0 (https://sites.google.com/view/lasy-seq/) (Additional file 1: Fig. S1). The library was sequenced using HiSeq 2500 (Illumina, San Diego, CA, USA) at Macrogen (Seoul, South Korea) with single-end sequencing lengths of 50 bp.

All obtained reads were trimmed using Trimmomatic version 0.36 [56] using the following parameters: TOPHRED33, ILLUMINACLIP: TruSeq3-SE.fa:2:30:10, LEADING:19, TRAILING:19, SLIDINGWINDOW:30:20, AVGQUAL:20, MINLEN:40, indicating that reads with more than 39 nucleotides and average quality scores over 19 were reported. Then, the trimmed reads were mapped onto the reference sequences of the IRGSP-1.0_transcript (2018-03-29) [57], the mitochondria (NC_011033.1) and the chloroplast (NC_001320.1) genomes, and the virus reference sequences, which were composed of complete genome sequences of 7457 viruses obtained from NCBI GenBank [6] using RSEM version 1.2.21 [58] and Bowtie version 1.1.1 [59] with default parameters. The reads per million (rpm) were calculated using the nuclear-encoded gene raw count data, excluding the genes encoding rRNA, as described by Kashima et al. [6]. The number of reads was 0.18–6.91 million per sample (Additional file 1: Fig. S1a; Additional file 2: Table S2).

### Sample swap correction

We corrected sample annotations by identifying outliers in the temperature regression on the transcriptome (Additional file 1: Fig. S2). When we made a statistical model that predicted the temperature inside the GCs from the transcriptome, the result implied that some sample IDs were wrongly annotated, seemingly due to samples being swapped at 96-well plate level during library processing for RNA-Seq. To evaluate the accuracy of the temperature prediction, we split the data into those from a specific plate (test data) and the others (training data). Using the training data, we parameterized a generalized linear model (GLM) with Gaussian distribution that predicted the temperature inside the GCs from the transcriptome. We used the lasso (least absolute shrinkage and selection operator) technique for sparse regression. We adopted the regularization parameter *λ* that minimized MAE when fitted to the training data. By applying this parameterized GLM to the test data, we predicted the temperature (we used “glmnet” package of R for this analysis). We repeated this procedure for all plates (Additional file 1: Fig. S2a). This analysis suggested that plates 9 and 11 (Additional file 2: Table S2) had been swapped during the lab work. It also indicated that plates 10 and 12 (Additional file 2: Table S2) had been similarly swapped. We re-annotated these samples, assuming that our inference was correct. We then repeated the same procedure (prediction of temperature). This second round of temperature prediction showed consistent, substantially improved accuracy and precision and did not imply a systematic sample interchange (Additional file 1: Fig. S2b). We thus assumed that the annotations used in this second prediction round were correct.

### Genotyping by sequence

We screened the genotypes of sequenced samples to correct possible sample swaps between cultivars (Additional file 1: Fig. S1b). Following the method of Kashima et al. [6], we genotyped samples by mRNA sequences. Briefly, we first compared sequences among samples and recorded the genotypes of each sample for all loci that were polymorphic in at least one of all possible sample pairs (247,645 loci). Among these loci, we used 26,746 loci known to show cultivar-specific SNP (a subset of previously established 1,427,878 loci with cultivar-specific SNP). Each locus of each sample was classified into one of four categories: Koshihikari genotype, Takanari genotype, unknown genotype (e.g., sequence error), or no sequence (number of sequenced loci were 4005–20,953, with 12,786.8 on average). We calculated the proportions of loci with cultivar-specific genotypes among sequenced loci (Additional file 1: Fig. S1b). More than 70% of loci matched the nominal cultivar in all samples, but one, about 50% of loci showed genotypes different from both cultivars (among 13,634 sequenced loci). We discarded this sample from the main analyses.

### Statistical analysis of transcriptome data in 73 GC conditions

R software version 4.1.0 was used for the t-SNE, PCA, the calculation of amplitude of gene expression, DEG analysis, Steel–Dwass tests, the calculation of Pearson’s correlation coefficient, co-expression gene network analysis, GO and KEGG enrichment tests [60]. A total of 17,742 genes in which the average log_2_(rpm + 1) was > 1 were used for the analyses (Additional file 1: Fig. S1c).

t-Distributed Stochastic Neighbor Embedding (t-SNE) was conducted using the R package “Rtsne” version 0.15 [61]. Principal component analysis was conducted using the prcomp() of the R. Violin plots were drawn using the R package “vioplot” version 0.4.0 [62].

The diel oscillation of each gene expression (log_2_(rpm + 1)) of each cultivar and condition were analyzed by the smooth.spline() of the R with the parameter spar set as 0.3 and intervals of 1.5 h. We defined the amplitude as the difference between the maximum and minimum values of the diel oscillation. A total of 12,770 genes whose average amplitudes of both Koshihikari and Takanari > 2 were used to compare the amplitudes between cultivar and conditions.

Differentially expressed genes (DEGs) were extracted by paired *t*-test in which samples of Koshihikari and Takanari at the same time point are regarded as paired samples. FDR was controlled using Benjamini and Hochberg’s method [63].

The Steel–Dwass test was performed using the pSDCFlig() function of the R package “NSM3,” version 1.19 [64] with 10,000 Monte Carlo simulations. Co-expression gene network analysis was conducted by the R package “WGCNA” version 1.70-3 [35]. An unsigned network was constructed using the adjacency and TOMsimilarity function with power = 14. Modules were detected using the cutreeDynamic function, with deepsplit = 4 and minClusterSize = 20. Similar modules were then merged using the mergeCloseModules function with cutHeight = 0.2. As a result, 5889 genes were assigned to 22 modules. Module Eigengene (ME), the first component in PCA of the gene expression profiles, was calculated for each module. The constructed networks were visualized using Cytoscape version 3.9.0 [65]. Genes with adjacency > 0.05 are shown. To explore the hub genes that can have a central role in the gene co-expression network regulation in module 5, we used NetworkAnalyzer in Cytoscape. Three genes with degree > 4 and betweenness centrality > 0.3 were extracted as hub genes.

Gene enrichment tests for GO and Kyoto Encyclopedia of Genes and Genomes (KEGG) [66] pathways were conducted using the R package “GO.db” version 3.13.0 [67] and “KEGGREST” version 1.32.0 [68], respectively, as described by Nagano et al. [7]. The FDR was controlled using Benjamini and Hochberg’s method [63] with FDR = 0.05.

### Selection of representative genes

We used microarray data of rice leaves sampled in paddy fields between June and September 2008 [8]. Briefly, rice seeds were sown in germination boxes and transplanted to paddy fields in Tsukuba, Japan, either 30 days or 14–15 days after germination. A total of 461 samples of the youngest fully expanded leaves were collected from rice plants at various developmental stages, ranging from the vegetative stage to the grain-filling stage, for DNA microarray analysis. Using these data, we performed affinity propagation clustering using 17,616 genes with expression greater than 2^5^ in more than 80% of the samples [69]. Five hundred clusters were obtained from the affinity propagation clustering using the R package “apcluster” [70]. The exemplars of 500 clusters were considered as representative genes.

Since the gene annotation used for the RNA-Seq in this study [57] was updated from those used in the microarray of Nagano et al. [8], we updated the gene ID of the genes based on the homology search using BLASTn for nucleotides [71]. Among the 17,616 genes, 16,634 genes exist in the gene annotation used for the RNA-Seq; 26 among 500 representative genes were obsoleted. We used the remaining 474 genes for the following analysis.

### Modeling gene expression dynamics

We used the R package “FIT” [16], which trains a model of gene expression dynamics as a function of meteorological time series, time of day, and days after transplanting. Gene expression (log_2_(rpm + 1)) was explained by the sum of sub-models (plant’s age, circadian clock, and environmental responses) and their interactions. Coefficients of the sub-models and phase parameters of the circadian clock were optimized using adaptive group LASSO [72, 73]. The sub-model of environmental response consisted of the sum of a gate function (diel changes in environmental responsiveness) applied to environmental variables over a period of time in the past. In the sub-model of environmental response, there are 7 parameters that cannot be optimized directly by adaptive group LASSO. Therefore, these parameters were optimized by grid search and the Nelder-Mead algorithm [74]. One of the temperature and radiation was chosen for each gene. Extending this approach to consider both temperature and radiation simultaneously would require a lot of computational effort and would make the optimization very unstable. For this reason, this paper did not consider models with both temperature and radiation.

Gene expression dynamics were modeled independently for the two cultivars, Koshihikari and Takanari. We trained the models using the GC data (Koshihikari: *n* = 584; Takanari: *n* = 583), field data (Koshihikari: *n* = 805; Takanari: *n* = 714), or a mixture of them (50% GC and 50% field). When model training failed in any data subsampling with any training data, such genes were excluded from subsequent analyses (genes were omitted independently between cultivars). The prediction performances of the models were tested using field data that was not included in the training data (615 data points for Koshihikari and 539 points for Takanari). We allowed the models to use the meteorological data up to 72 h ago. The field data were obtained according to the method as previously described [6]. The field data used for model training were collected from rice plants grown in paddy fields in Takatsuki, Japan (34°51′19″N, 135°37′51″E) from May 25 to September 29, 2015, and in Kizugawa, Japan (34°44′03″N, 135°50′18″E) from May 26 to September 30, 2016. The data used for model evaluation were obtained from rice plants grown in a paddy field in Kizugawa in 2017. During the sampling period, the average daily mean temperatures were 25.0 °C in 2015, 25.0 °C in 2016, and 24.9 °C in 2017; the highest temperatures were 37.8 °C, 36.7 °C, and 36.9 °C, and the lowest were 14.0 °C, 9.6 °C, and 11.3 °C, respectively.

## Supplementary Information

Additional file 1: Fig. S1 Workflow of the RNA-Seq data preprocessing. Fig. S2 Swap correction. Fig. S3 t-Distributed Stochastic Neighbor Embedding (t-SNE) visualization showing clusters of transcriptomes of each sample. Fig. S4 Distribution of expression amplitudes across 73 conditions. Fig. S5 Normalized expressions of eigengenes of each module. Fig. S6 Expression levels of a gene encoding *Hsp 70* (*Os01g0840100*). Fig. S7 The number of times temperature or radiation (or neither) was chosen as the predictor of gene expression (Takanari). Fig. S8 Prediction performances of gene expression models trained with different data sets in Takanari. Fig. S9 Prediction performances of gene expression models trained with different data sets in Koshihikari.

Additional file 2: Table S1 73 environmental conditions used in this study. Table S2 Sample attributes used in this study. Table S3 Enriched gene ontology in each module. Table S4 Enriched KEGG pathway in each module. Table S5 List of genes in module 5. Table S6 List of genes with positive correlation in expression with air temperature. Table S7 List of genes with negative correlation in expression with air temperature. Table S8 Comparison of the relative frequency with which radiation and temperature are selected as predictors for representative genes across models in Koshihikari. Table S9 Enriched gene ontology terms and KEGG pathways in gene clusters whose expression of the representative genes was more frequently predicted by radiation or temperature than by the other predictor across models in Koshihikari. Table S10 Comparison of the relative frequency with which radiation and temperature are selected as predictors for representative genes across models in Takanari. Table S11 Enriched gene ontology terms and KEGG pathways in gene clusters whose expression of the representative genes was more frequently predicted by radiation or temperature than by the other predictor across models in Takanari. Table S12 List of the representative genes whose predictors were temperature in more than 80% of the field models but radiation in more than 80% of the mixed models. Table S13 Enriched gene ontology terms and KEGG pathways in gene clusters whose expression of the representative genes was predicted by temperature in more than 80% of the field models but by radiation in more than 80% of the mixed models. Table S14 List of the representative genes whose predictors were radiation in more than 80% of the field models and the mixed models. Table S15 Enriched gene ontology terms and KEGG pathways in gene clusters whose expression of the representative genes was predicted by radiation in more than 80% of the field models and the mixed models. Table S16 List of the representative genes whose predictors were temperature in more than 80% of the field models and the mixed models. Table S17 Enriched gene ontology terms and KEGG pathways in gene clusters whose expression of the representative genes was predicted by temperature in more than 80% of the field models and the mixed models. Table S18 List of the representative genes for which the prediction performance was better in the GC or mixed model than in the field model in Koshihikari. Table S19 Gene ontology terms and KEGG pathways enriched in the genes with better prediction performance in the GC or mixed model than in the field model in Koshihikari. Table S20 List of the representative genes for which the prediction performance was better in the GC or mixed model than in the field model in Takanari. Table S21 Gene ontology terms and KEGG pathways enriched in the genes with better prediction performance in the GC or mixed model than in the field model in Takanari.

## Data Availability

The scripts used in this study are available on GitHub at https://github.com/naganolab/GC_73_conditions [75] and on Zenodo at https://doi.org/10.5281/zenodo.15811371 [76] under the MIT license. The raw sequencing data generated in this study are available in the Sequence Read Archive (SRA) under accession number PRJNA1074001 [77]. The published rice reference genome data used in this study were downloaded from the Rice Annotation Project Database [57, 78] and from NCBI under accession numbers NC_011033.1 [79] and NC_001320.1 [80].

## References

[1] Panter PE, Muranaka T, Cuitun-Coronado D, Graham CA, Yochikawa A, Kudoh H, et al. Circadian regulation of the plant transcriptome under natural conditions. Front Genet. 2019;10:1–12. 31850080 10.3389/fgene.2019.01239 PMC6895068

[2] Poorter H, Fiorani F, Pieruschka R, Wojciechowski T, van der Putten WH, Kleyer M, et al. Pampered inside, pestered outside? Differences and similarities between plants growing in controlled conditions and in the field. New Phytol. 2016;212:838–55. 27783423 10.1111/nph.14243

[3] Alexandersson E, Jacobson D, Vivier MA, Weckwerth W, Andreasson E. Field-omics-understanding large-scale molecular data from field crops. Front Plant Sci. 2014;5:1–6. 10.3389/fpls.2014.00286 PMC4064663 24999347

[4] Nishio H, Buzas DM, Nagano AJ, Iwayama K, Ushio M, Kudoh H. Repressive chromatin modification underpins the long-term expression trend of a perennial flowering gene in nature. Nat Commun. 2020;11:2065. 32358518 10.1038/s41467-020-15896-4 PMC7195410

[5] Nishio H, Nagano AJ, Ito T, Suzuki Y, Kudoh H. Seasonal plasticity and diel stability of H3K27me3 in natural fluctuating environments. Nat Plants. 2020;6:1091–7. 32868888 10.1038/s41477-020-00757-1

[6] Kashima M, Sakamoto RL, Saito H, Ohkubo S, Tezuka A, Deguchi A, et al. Genomic basis of transcriptome dynamics in rice under field conditions. Plant Cell Physiol. 2021;62:1436–45. 34131748 10.1093/pcp/pcab088 PMC8600290

[7] Nagano AJ, Kawagoe T, Sugisaka J, Honjo MN, Iwayama K, Kudoh H. Annual transcriptome dynamics in natural environments reveals plant seasonal adaptation. Nat Plants. 2019;5:74–83. 30617252 10.1038/s41477-018-0338-z

[8] Nagano AJ, Sato Y, Mihara M, Antonio BA, Motoyama R, Itoh H, et al. Deciphering and prediction of transcriptome dynamics under fluctuating field conditions. Cell. 2012;151:1358–69. 23217716 10.1016/j.cell.2012.10.048

[9] Matsuzaki J, Kawahara Y, Izawa T. Punctual transcriptional regulation by the rice circadian clock under fluctuating field conditions. Plant Cell. 2015;27:633–48. 25757473 10.1105/tpc.114.135582 PMC4558668

[10] Takehisa H, Ando F, Takara Y, Ikehata A, Sato Y. Transcriptome and hyperspectral profiling allows assessment of phosphorus nutrient status in rice under field conditions. Plant Cell Environ. 2022;45:1507–19. 35128701 10.1111/pce.14280

[11] Sato Y, Antonio B, Namiki N, Motoyama R, Sugimoto K, Takehisa H, et al. Field transcriptome revealed critical developmental and physiological transitions involved in the expression of growth potential in japonica rice. BMC Plant Biol. 2011;11:10. 21226959 10.1186/1471-2229-11-10 PMC3031230

[12] Takehisa H, Sato Y. Transcriptome monitoring visualizes growth stage-dependent nutrient status dynamics in rice under field conditions. Plant J. 2019;97:1048–60. 30481387 10.1111/tpj.14176

[13] Michael TP. Time of day analysis over a field grown developmental time course in rice. Plants. 2023;12:166. 10.3390/plants12010166 PMC9823482 36616295

[14] Matsunami M, Murai-Hatano M, Kuwagata T, Matsushima U, Hashida Y, Tominaga Y, et al. Transcriptome dynamics of rice in natura: response of above and below ground organs to microclimate. Plant Cell Environ. 2023;46:1176–94. 36111882 10.1111/pce.14439

[15] Plessis A, Hafemeister C, Wilkins O, Gonzaga ZJ, Meyer RS, Pires I, et al. Multiple abiotic stimuli are integrated in the regulation of rice gene expression under field conditions. Elife. 2015;4: e08411. 26609814 10.7554/eLife.08411 PMC4718725

[16] Iwayama K, Aisaka Y, Kutsuna N, Nagano AJ. FIT: statistical modeling tool for transcriptome dynamics under fluctuating field conditions. Bioinformatics. 2017;33:1672–80. 28158396 10.1093/bioinformatics/btx049 PMC5447243

[17] Matsubara S. Growing plants in fluctuating environments: why bother? J Exp Bot. 2018;69:4651–4. 30307518 10.1093/jxb/ery312 PMC6137991

[18] Hashida Y, Tezuka A, Nomura Y, Kamitani M, Kashima M, Kurita Y, et al. Fillable and unfillable gaps in plant transcriptome under field and controlled environments. Plant Cell Environ. 2022;45:2410–27. 35610174 10.1111/pce.14367 PMC9544781

[19] Kurita Y, Takimoto H, Kamitani M, Hashida Y, Kashima M, Tezuka A, et al. Integration of short- and long-term responses to environmental stimuli shape seasonal transcriptome dynamics. BioRxiv. 2022. Available from: http://biorxiv.org/lookup/doi/10.1101/2021.08.02.454700.

[20] Yamaguchi N, Matsubara S, Yoshimizu K, Seki M, Hamada K, Kamitani M, et al. H3K27me3 demethylases alter HSP22 and HSP17.6C expression in response to recurring heat in Arabidopsis. Nat Commun. 2021;12:3480. 34108473 10.1038/s41467-021-23766-w PMC8190089

[21] Itoh H, Yamashita H, Wada KC, Yonemaru J. Real-time emulation of future global warming reveals realistic impacts on the phenological response and quality deterioration in rice. Proc Natl Acad Sci U S A. 2024;121: e2316497121. 38739807 10.1073/pnas.2316497121 PMC11126993

[22] Tanaka Y, Adachi S, Yamori W. Natural genetic variation of the photosynthetic induction response to fluctuating light environment. Curr Opin Plant Biol. 2019;49:52–9. 31202005 10.1016/j.pbi.2019.04.010

[23] Acebron K, Matsubara S, Jedmowski C, Emin D, Muller O, Rascher U. Diurnal dynamics of nonphotochemical quenching in Arabidopsis *npq* mutants assessed by solar-induced fluorescence and reflectance measurements in the field. New Phytol. 2021;229:2104–19. 33020945 10.1111/nph.16984

[24] Niedermaier S, Schneider T, Bahl MO, Matsubara S, Huesgen PF. Photoprotective acclimation of the Arabidopsis thaliana leaf proteome to fluctuating light. Front Genet. 2020;11:154. 32194630 10.3389/fgene.2020.00154 PMC7066320

[25] Song YH, Kubota A, Kwon MS, Covington MF, Lee N, Taagen ER, et al. Molecular basis of flowering under natural long-day conditions in Arabidopsis. Nat Plants. 2018;4:824–35. 30250277 10.1038/s41477-018-0253-3 PMC6195122

[26] Kim H, Kang HW, Hwang DY, Lee N, Kubota A, Imaizumi T, et al. Low temperature-mediated repression and far-red light-mediated induction determine morning FLOWERING LOCUS T expression levels. J Integr Plant Biol. 2024;66:103–20. 38088490 10.1111/jipb.13595 PMC10829767

[27] Lee N, Ozaki Y, Hempton AK, Takagi H, Purusuwashi S, Song YH, et al. The FLOWERING LOCUS T gene expression is controlled by high-irradiance response and external coincidence mechanism in long days in Arabidopsis. New Phytol. 2023;239:208–21. 37084001 10.1111/nph.18932 PMC10244125

[28] Annunziata MG, Apelt F, Carillo P, Krause U, Feil R, Mengin V, et al. Getting back to nature: a reality check for experiments in controlled environments. J Exp Bot. 2017;68:4463–77. 28673035 10.1093/jxb/erx220 PMC5853417

[29] Annunziata MG, Apelt F, Carillo P, Krause U, Feil R, Koehl K, et al. Response of Arabidopsis primary metabolism and circadian clock to low night temperature in a natural light environment. J Exp Bot. 2018;69:4881–95. 30053131 10.1093/jxb/ery276 PMC6137998

[30] Adachi S, Tanaka Y, Miyagi A, Kashima M, Tezuka A, Toya Y, et al. High-yielding rice Takanari has superior photosynthetic response to a commercial rice Koshihikari under fluctuating light. J Exp Bot. 2019;70:5287–97. 31257443 10.1093/jxb/erz304 PMC6793460

[31] Liu X, Lu T, Yu S, Li Y, Huang Y, Huang T, et al. A collection of 10,096 indica rice full-length cDNAs reveals highly expressed sequence divergence between *Oryza sativa* indica and japonica subspecies. Plant Mol Biol. 2007;65:403–15. 17522955 10.1007/s11103-007-9174-7

[32] Jung KH, Gho HJ, Giong HK, Chandran AKN, Nguyen QN, Choi H, et al. Genome-wide identification and analysis of Japonica and Indica cultivar-preferred transcripts in rice using 983 Affymetrix array data. Rice. 2013;6:1–13. 24280533 10.1186/1939-8433-6-19 PMC4883688

[33] Zhou Q, Fu H, Yang D, Ye C, Zhu S, Lin J, et al. Differential alternative polyadenylation contributes to the developmental divergence between two rice subspecies, japonica and indica. Plant J. 2019;98:260–76. 30570805 10.1111/tpj.14209

[34] Mackill DJ, Lei X. Genetic variation for traits related to temperate adaptation of rice cultivars. Crop Sci. 1997;37:1340–6.

[35] Langfelder P, Horvath S. WGCNA: an R package for weighted correlation network analysis. BMC Bioinformatics. 2008;9:559. 19114008 10.1186/1471-2105-9-559 PMC2631488

[36] Kitazawa N, Shomura A, Mizubayashi T, Ando T, Nagata K, Hayashi N, et al. Rapid DNA-genotyping system targeting ten loci for resistance to blast disease in rice. Breed Sci. 2019;69:68–83. 31086485 10.1270/jsbbs.18143 PMC6507720

[37] Qian D, Xiong S, Li M, Tian L, Qu LQ. OsFes1C, a potential nucleotide exchange factor for OsBiP1, is involved in the ER and salt stress responses. Plant Physiol. 2021;187:396–408. 34618140 10.1093/plphys/kiab263 PMC8418431

[38] Qi Y, Wang H, Zou Y, Liu C, Liu Y, Wang Y, et al. Over-expression of mitochondrial heat shock protein 70 suppresses programmed cell death in rice. FEBS Lett. 2011;585:231–9. 21130768 10.1016/j.febslet.2010.11.051

[39] Ahn JC, Kim DW, You YN, Seok MS, Park JM, Hwang H, et al. Classification of rice (*Oryza sativa* L. Japonica nipponbare) immunophilins (FKBPs, CYPs) and expression patterns under water stress. BMC Plant Biol. 2010;10:253. 21087465 10.1186/1471-2229-10-253 PMC3012604

[40] Pedersen DS, Merkle T, Marktl B, Lildballe DL, Antosch M, Bergmann T, et al. Nucleocytoplasmic distribution of the arabidopsis chromatin-associated HMGB2/3 and HMGB4 proteins. Plant Physiol. 2010;154:1831–41. 20940346 10.1104/pp.110.163055 PMC2996034

[41] Min MK, Choi EH, Kim JA, Yoon IS, Han S, Lee Y, et al. Two clade A phosphatase 2Cs expressed in guard cells physically interact with abscisic acid signaling components to induce stomatal closure in rice. Rice. 2019;12:1–13. 31134357 10.1186/s12284-019-0297-7 PMC6536566

[42] MacKinnon KJM, Cole BJ, Yu C, Coomey JH, Hartwick NT, Remigereau MS, et al. Changes in ambient temperature are the prevailing cue in determining Brachypodium distachyon diurnal gene regulation. New Phytol. 2020;227:1709–24. 32112414 10.1111/nph.16507

[43] Desai JS, Lawas LMF, Valente AM, Leman AR, Grinevich DO, Jagadish SVK, et al. Warm nights disrupt transcriptome rhythms in field-grown rice panicles. Proc Natl Acad Sci U S A. 2021;118:1–12. 10.1073/pnas.2025899118 PMC8237568 34155145

[44] Filichkin SA, Breton G, Priest HD, Dharmawardhana P, Jaiswal P, Fox SE, et al. Global profiling of rice and poplar transcriptomes highlights key conserved circadian-controlled pathways and cis-regulatory modules. PLoS ONE. 2011;6: e16907. 21694767 10.1371/journal.pone.0016907 PMC3111414

[45] Michael TP, Salomé PA, McClung CR. Two Arabidopsis circadian oscillators can be distinguished by differential temperature sensitivity. Proc Natl Acad Sci U S A. 2003;100:6878–83. 12736379 10.1073/pnas.1131995100 PMC164540

[46] Bours R, van Zanten M, Pierik R, Bouwmeester H, van der Krol A. Antiphase light and temperature cycles affect PHYTOCHROME B-controlled ethylene sensitivity and biosynthesis, limiting leaf movement and growth of *Arabidopsis*. Plant Physiol. 2013;163:882–95. 23979970 10.1104/pp.113.221648 PMC3793065

[47] Shimizu H. Effect of day and night temperature alternations on plant morphogenesis. Environ Control Biol. 2007;45:259–65.

[48] Azodi CB, Pardo J, VanBuren R, de Los CG, Shiu SH. Transcriptome-based prediction of complex traits in maize. Plant Cell. 2020;32:139–51. 31641024 10.1105/tpc.19.00332 PMC6961623

[49] Li Z, Gao N, Martini JWR, Simianer H. Integrating gene expression data into genomic prediction. Front Genet. 2019;10:1–11. 30858865 10.3389/fgene.2019.00126 PMC6397893

[50] Hershberger J, Tanaka R, Wood JC, Kaczmar N, Wu D, Hamilton JP, et al. Transcriptome-wide association and prediction for carotenoids and tocochromanols in fresh sweet corn kernels. Plant Genome. 2022;15:1–16. 10.1002/tpg2.20197 PMC12807274 35262278

[51] Xu Y, Zhang X, Li H, Zheng H, Zhang J, Olsen MS, et al. Smart breeding driven by big data, artificial intelligence, and integrated genomic-enviromic prediction. Mol Plant. 2022;15:1664–95. 36081348 10.1016/j.molp.2022.09.001

[52] Wang K, Abid MA, Rasheed A, Crossa J, Hearne S, Li H. DNNGP, a deep neural network-based method for genomic prediction using multi-omics data in plants. Mol Plant. 2023;16:279–93. 36366781 10.1016/j.molp.2022.11.004

[53] De Meyer S, Cruz DF, De Swaef T, Lootens P, De Block J, Bird K, et al. Predicting yield of individual field-grown rapeseed plants from rosette-stage leaf gene expression. PLoS Comput Biol. 2023;19: e1011161. 37253069 10.1371/journal.pcbi.1011161 PMC10256231

[54] Peng B, Guan K, Tang J, Ainsworth EA, Asseng S, Bernacchi CJ, et al. Towards a multiscale crop modelling framework for climate change adaptation assessment. Nat Plants. 2020;6:338–48. 32296143 10.1038/s41477-020-0625-3

[55] Kamitani M, Kashima M, Tezuka A, Nagano AJ. Lasy-Seq: a high-throughput library preparation method for RNA-Seq and its application in the analysis of plant responses to fluctuating temperatures. Sci Rep. 2019;9:7091. 31068632 10.1038/s41598-019-43600-0 PMC6506593

[56] Bolger AM, Lohse M, Usadel B. Trimmomatic: a flexible trimmer for Illumina sequence data. Bioinformatics. 2014;30:2114–20. 24695404 10.1093/bioinformatics/btu170 PMC4103590

[57] Kawahara Y, de la Bastide M, Hamilton JP, Kanamori H, Mccombie WR, Ouyang S, et al. Improvement of the *Oryza sativa* Nipponbare reference genome using next generation sequence and optical map data. Rice. 2013;6:4. 24280374 10.1186/1939-8433-6-4 PMC5395016

[58] Li B, Deway CN. RSEM: accurate transcript quantification from RNA-Seq data with or without a reference genome. BMC Bioinformatics. 2011;12:323. 21816040 10.1186/1471-2105-12-323 PMC3163565

[59] Langmead B, Trapnell C, Pop M, Salzberg SL. Ultrafast and memory-efficient alignment of short DNA sequences to the human genome. Genome Biol. 2009;10:R25. 19261174 10.1186/gb-2009-10-3-r25 PMC2690996

[60] R Core Team. R: a language and environment for statistical computing. Vienna, Austria: R Foundation for Statistical Computing; 2021. Available from: https://www.r-project.org/.

[61] Krijthe JH. Rtsne: t-distributed stochastic neighbor embedding using Barnes-Hut implementation. R Package version 0.15. 2015; Available from: https://github.com/jkrijthe/Rtsne.

[62] Adler D, Kelly ST, Elliot T, Adamson J. vioplot: violin plot. R package version 0.4.0. 2022. Available from: https://github.com/TomKellyGenetics/vioplot.

[63] Benjamini Y, Hochberg Y. Controlling the false discovery rate: a practical and powerful approach to multiple testing. J R Stat Soc Ser B. 1995;57:289–300.

[64] Schneider G, Chicken E, Becvarik R. NSM3: functions and datasets to accompany Hollander, Wolfe, and Chicken - nonparametric statistical methods, third edition.. 2024. Available from: https://cran.r-project.org/package=NSM3.

[65] Shannon P, Markiel A, Ozier O, Baliga NS, Wang JT, Ramage D, et al. Cytoscape: a software environment for integrated models. Genome Res. 2003;13:2498–504. 14597658 10.1101/gr.1239303 PMC403769

[66] Kanehisa M, Goto S. KEGG: Kyoto Encyclopedia of Genes and Genomes. Nucleic Acids Res. 2000;28:27–30. 10592173 10.1093/nar/28.1.27 PMC102409

[67] Carlson M. GO.db: a set of annotation maps describing the entire Gene Ontology. 2018.

[68] Tenenbaum D, Bioconductor package maintainer. KEGGREST: client-side REST access to the Kyoto Encyclopedia of Genes and Genomes (KEGG). 2021.

[69] Eiju D, Hashida Y, Maeda T, Iwayama K, Nagano AJ. Simulation study of factors affecting the accuracy of transcriptome models under complex environments. Plant Mol Biol. 2025;115:52. 40153098 10.1007/s11103-025-01578-6

[70] Bodenhofer U, Kothmeier A, Hochreiter S. Apcluster: an R package for affinity propagation clustering. Bioinformatics. 2011;27:2463–4. 21737437 10.1093/bioinformatics/btr406

[71] Camacho C, Coulouris G, Avagyan V, Ma N, Papadopoulos J, Bealer K, et al. BLAST+: architecture and applications. BMC Bioinformatics. 2009;10:421. 20003500 10.1186/1471-2105-10-421 PMC2803857

[72] Zou H. The adaptive lasso and its oracle properties. 2006;101:1418–29.

[73] Wang H, Leng C. A note on adaptive group lasso. Comput Stat Data Anal. 2008;52:5277–86.

[74] Nelder JA, Mead R. A simplex method for function minimization. Comput J. 1965;7:308–13.

[75] Hashida Y, Kyogoku D, Nagano AJ. R source code and data. GitHub. Available from: https://github.com/naganolab/GC_73_conditions. 2025.

[76] Hashida Y, Kyogoku D, Nagano AJ. 2025. R source code and data. Zenodo. 10.5281/zenodo.15811371.

[77] Hashida Y, Kyogoku D, Tanaka SE, Nagano AJ. Diurnal transcriptome of rice leaves of two cultivars under 73 controlled conditions of different temperature and day length. PRJNA1074001. Sequence Read Archive. https://www.ncbi.nlm.nih.gov/bioproject/PRJNA1074001/. 2025.

[78] Rice Annotation Project Database. IRGSP-1.0_transcript (2018–03–29). http://rapdb.dna.affrc.go.jp/.

[79] Oryza sativa Japonica Group mitochondrion, complete genome. National Center for Biotechnology Information. https://www.ncbi.nlm.nih.gov/nuccore/NC_011033.1. Cited 2025 Jul 5.

[80] Oryza sativa Japonica Group plastid, complete genome. National Center for Biotechnology Information. https://www.ncbi.nlm.nih.gov/nuccore/NC_001320.1. Cited 2025 Jul 5.

