# Supplementary material for: Field-crop transcriptome models are enhanced by measurements in systematically controlled environments
Source: Genome Biol. 2025 Jul 28;26:225. doi: 10.1186/s13059-025-03690-8 (PMC12302906; doi:10.1186/s13059-025-03690-8)
Supplement: Supplementary file 1 — Additional file 1: Fig. S1 Workflow of the RNA-Seq data preprocessing. Fig. S2 Swap correction. Fig. S3 t-Distributed Stochastic Neighbor Embedding (t-SNE) visualization showing clusters of transcriptomes of each sample. Fig. S4 Distribution of expression amplitudes across 73 conditions. Fig. S5 Normalized expressions of eigengenes of each module. Fig. S6 Expression levels of a gene encoding Hsp 70 (Os01g0840100). Fig. S7 The number of times temperature or radiation (or neither) was chosen as the predictor of gene expression (Takanari). Fig. S8 Prediction performances of gene expression models trained with different data sets in Takanari. Fig. S9 Prediction performances of gene expression models trained with different data sets in Koshihikari. [file 13059_2025_3690_MOESM1_ESM.docx]

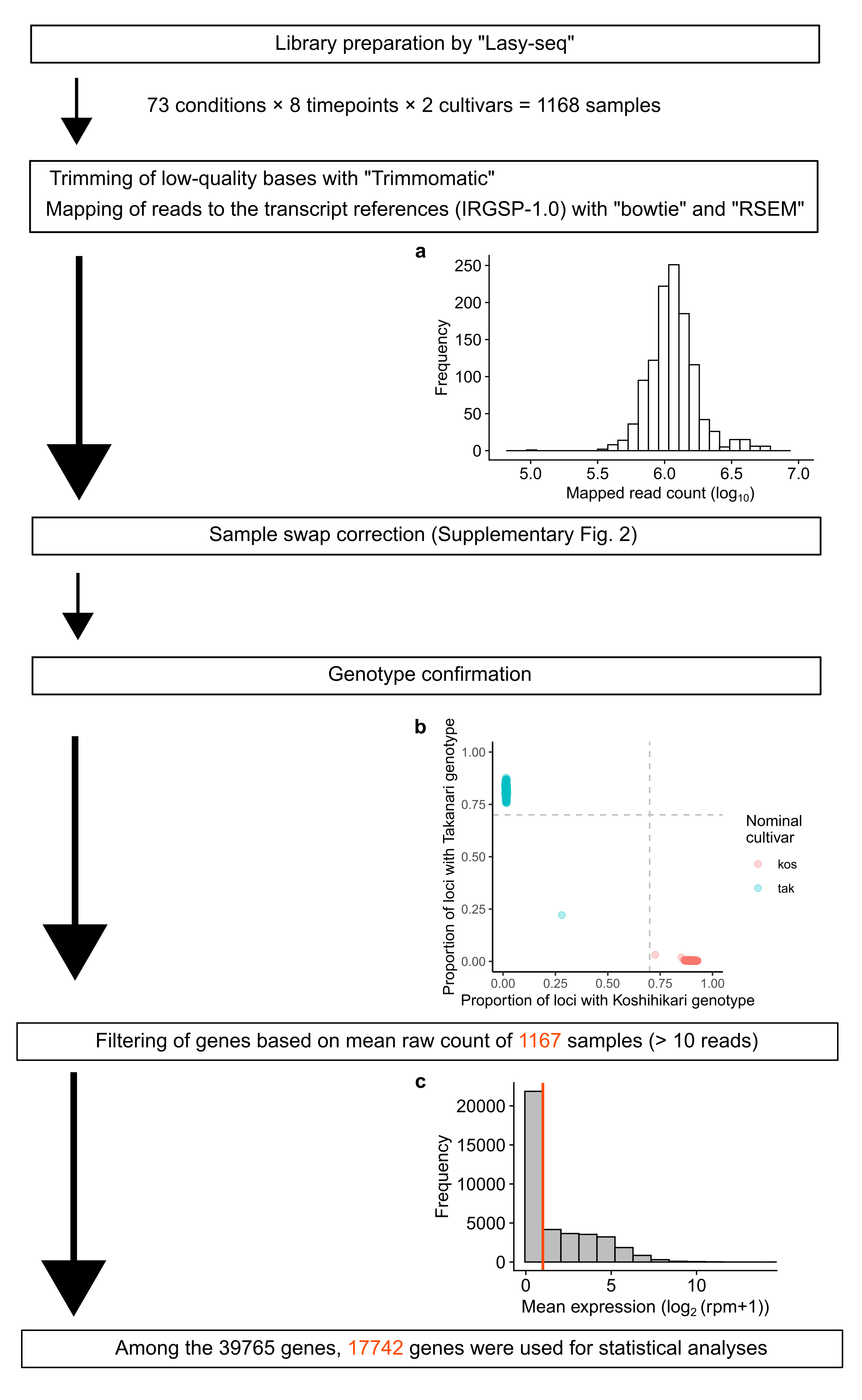
**Fig. S1 Workflow of the RNA-Seq data preprocessing.**

**a**, Histogram of the mapped read counts used for the calculation of rpm for each sample. **b**, Genotyping by sequence. Position of each sample in the two-dimensional genotype space. One sample was omitted based on the genotyping. **c**, Histogram of the mean expression for each gene. After filtering, 17,742 genes were used for the analyses. The red line represents log_2_(rpm + 1) = 1, which is the threshold used to define expressed genes in our analyses. Genes with values above this threshold are considered expressed.


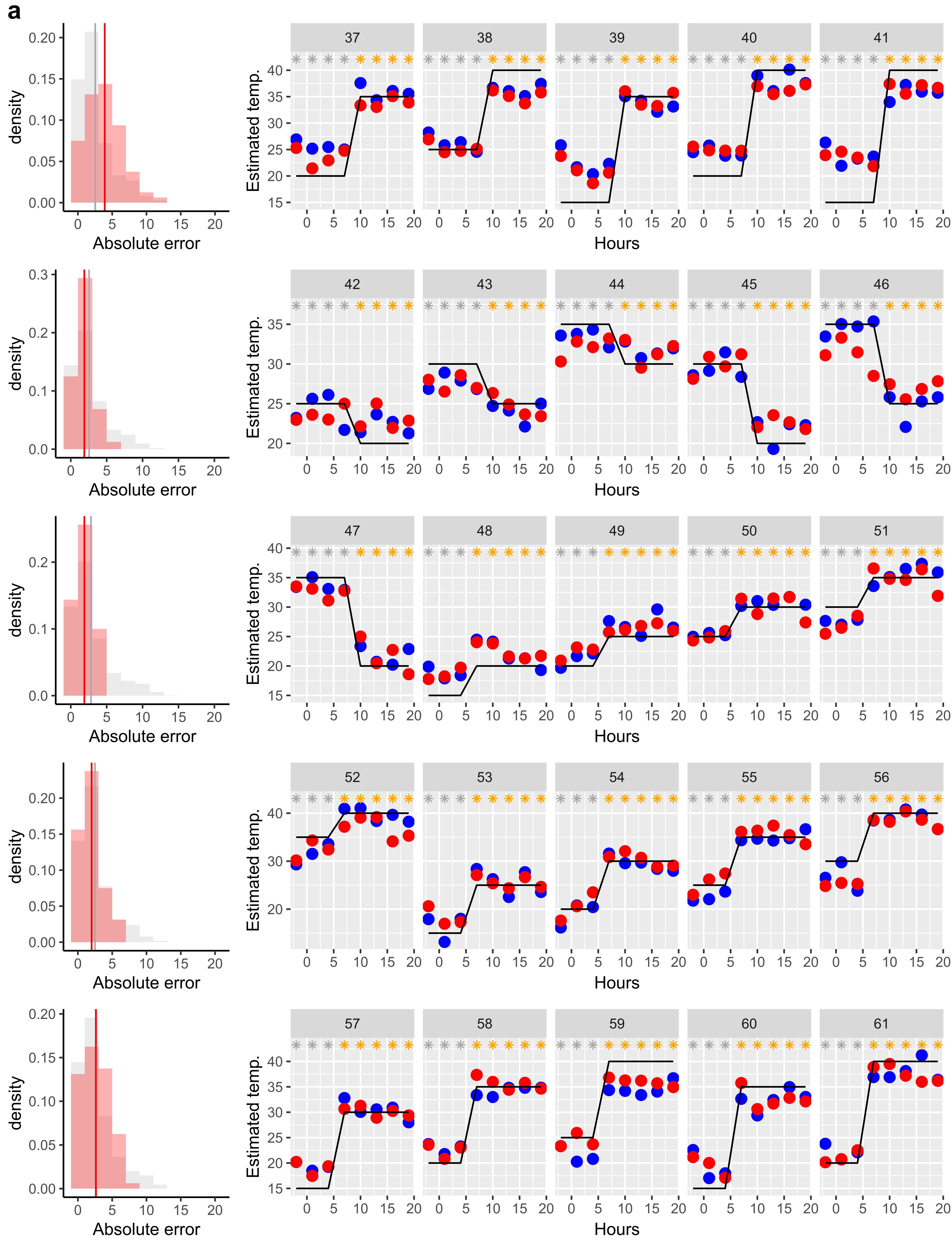


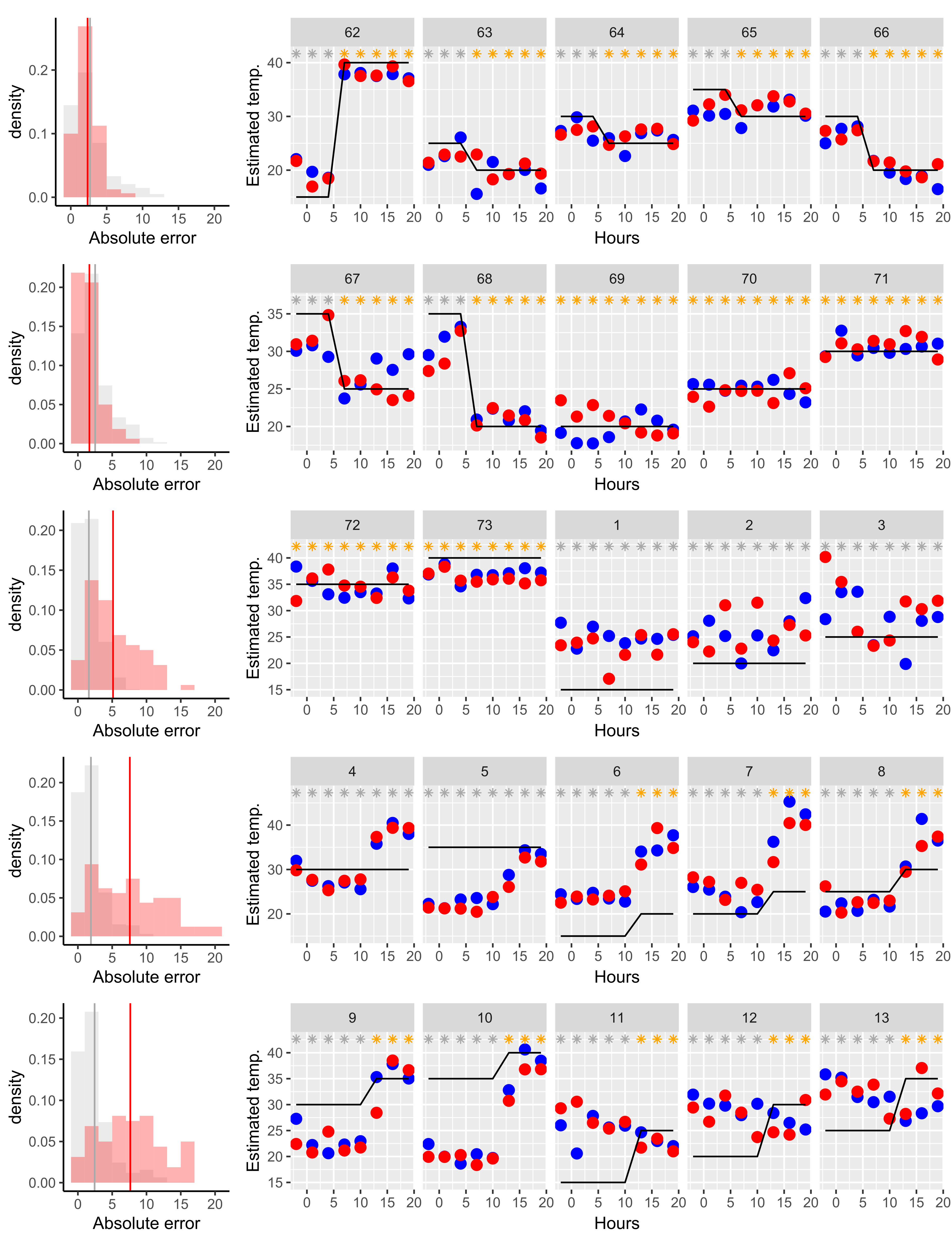


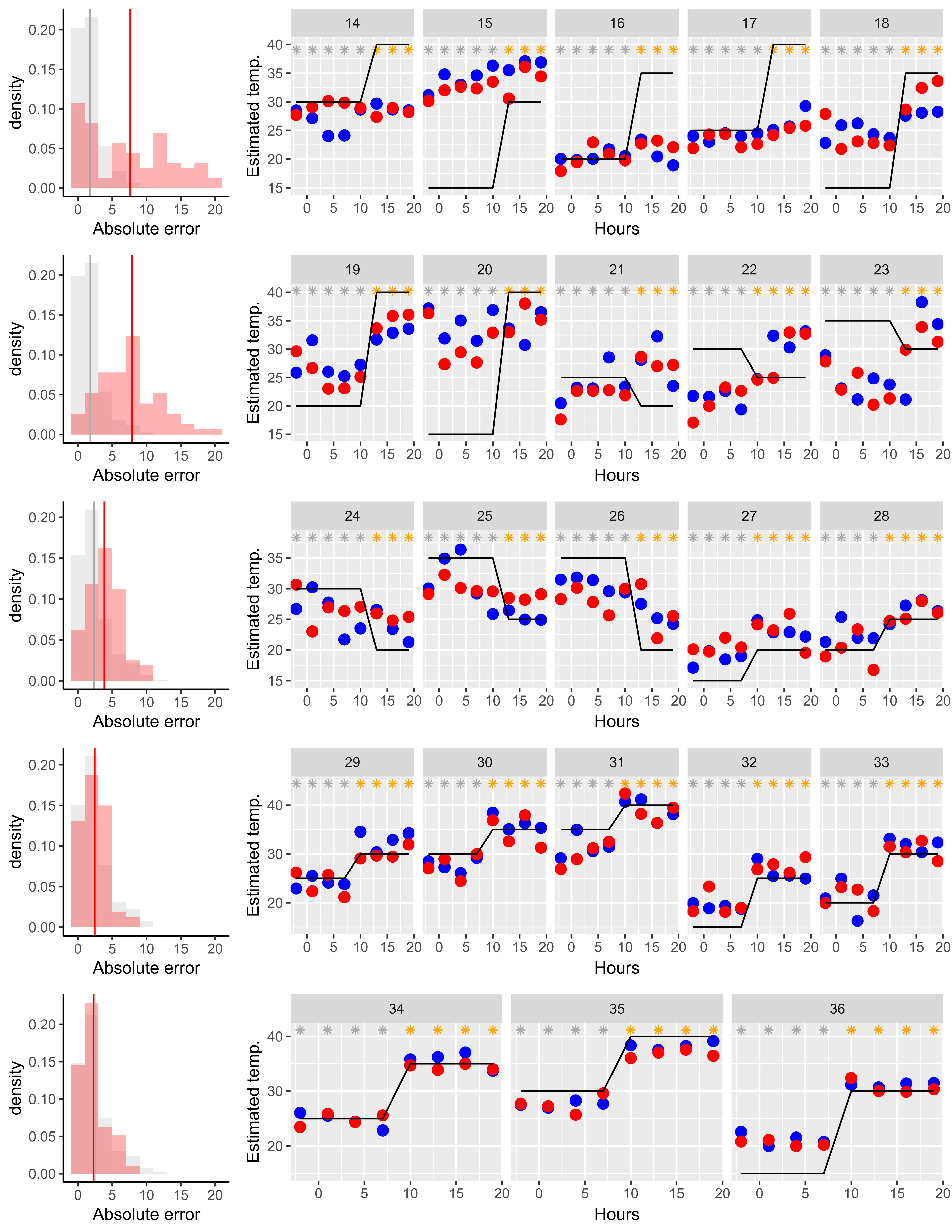


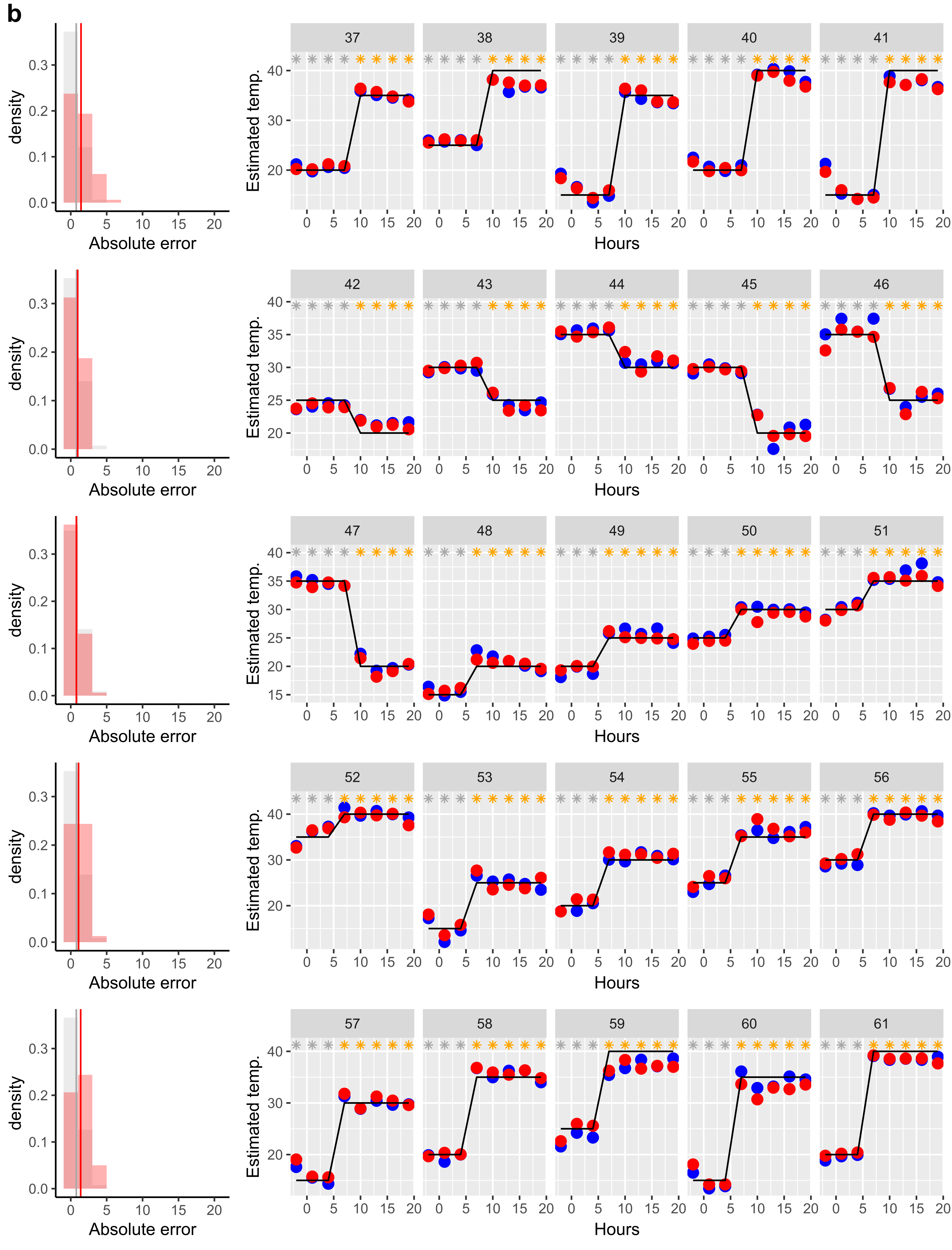


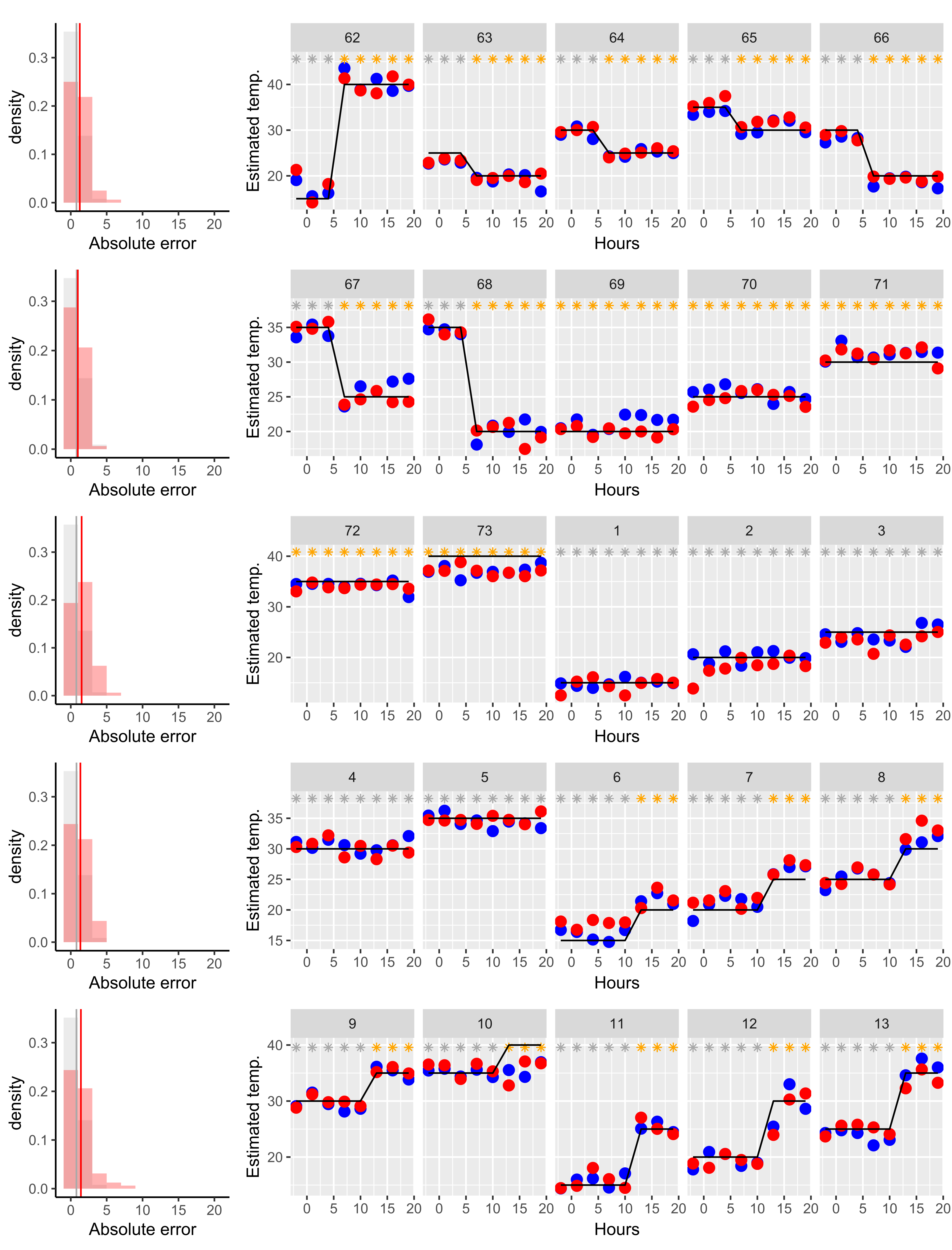


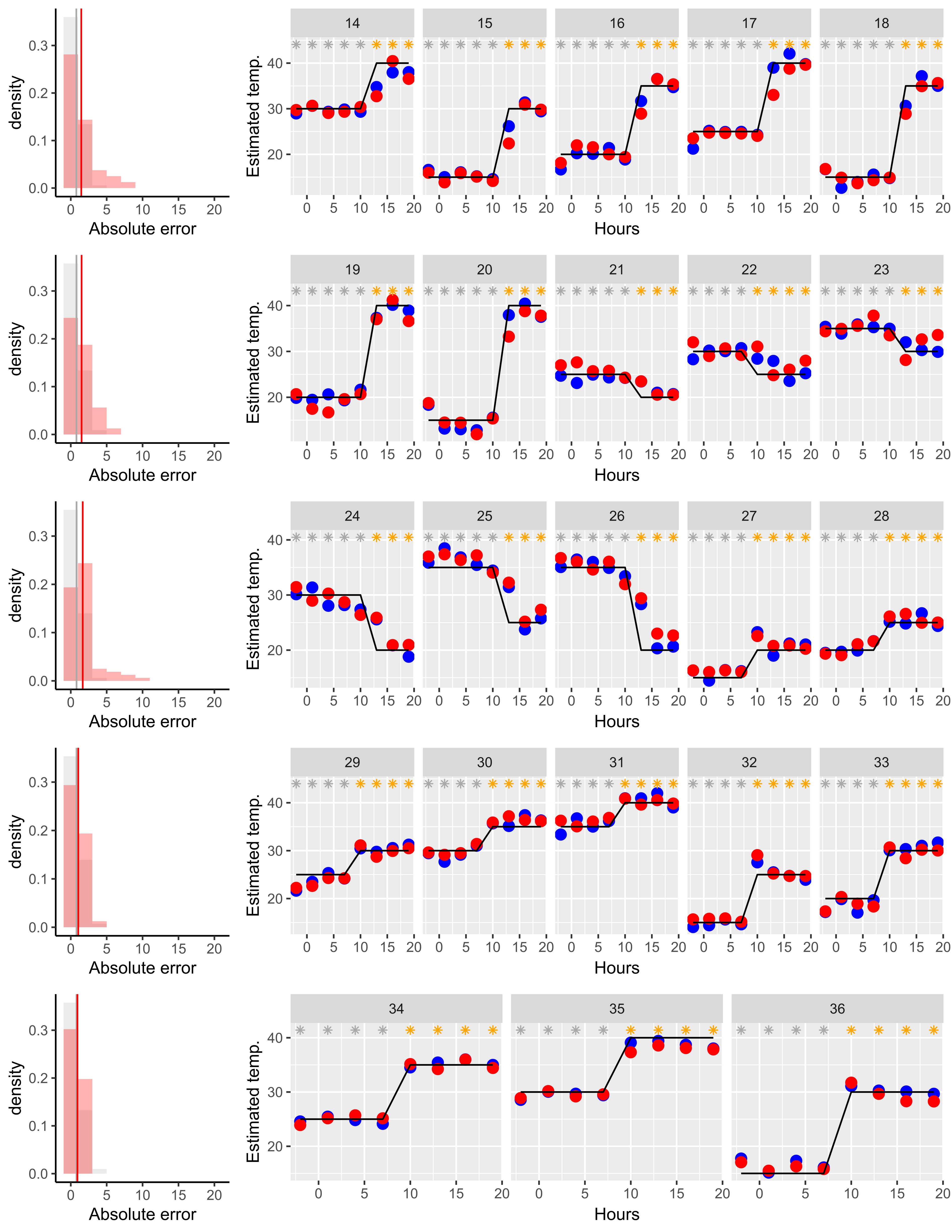


**Fig. S2 Swap correction.**

Performance of statistical models predicting the temperature inside the GCs during the first (**a**) and second (**b**) rounds. Each panel corresponds to a 96-well plate. In each panel, time series plots represent the model predictions (blue: Koshihikari, red: Takanari), and solid lines represent GC settings. Grey and orange asterisks represent light-dark cycles (grey: dark, orange: light). The numbers on the top of time series plots are experimental batches. Histograms show the absolute error distributions (red: that for the test data, grey: that for the training data). Vertical lines on the histograms represent mean absolute errors.

**
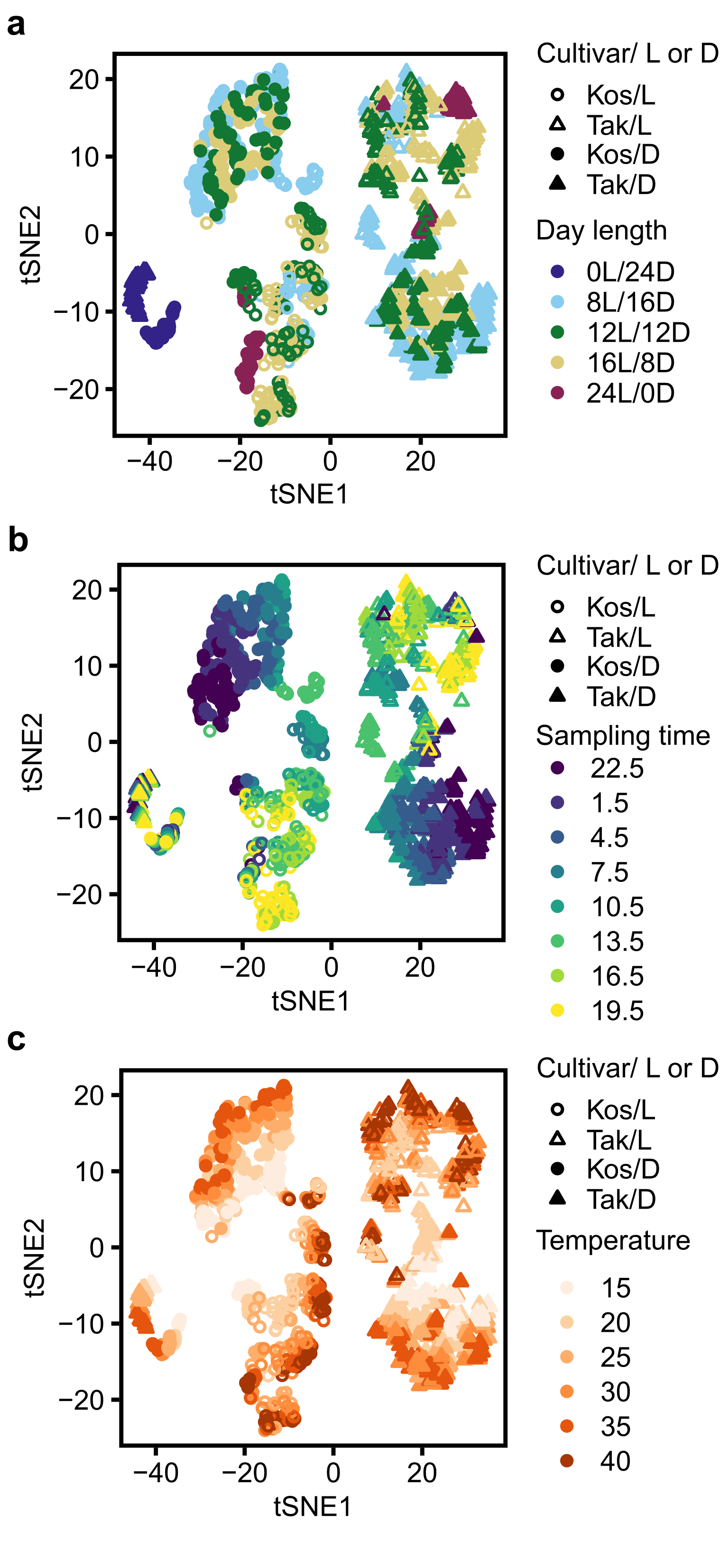
Fig. S3 t-Distributed Stochastic Neighbor Embedding (t-SNE) visualization showing clusters of transcriptomes of each sample.**

After clustering by t-SNE**,** the samples are colored by (**a**) light period length, (**b**) light period temperature, and (**c**) dark period temperature.


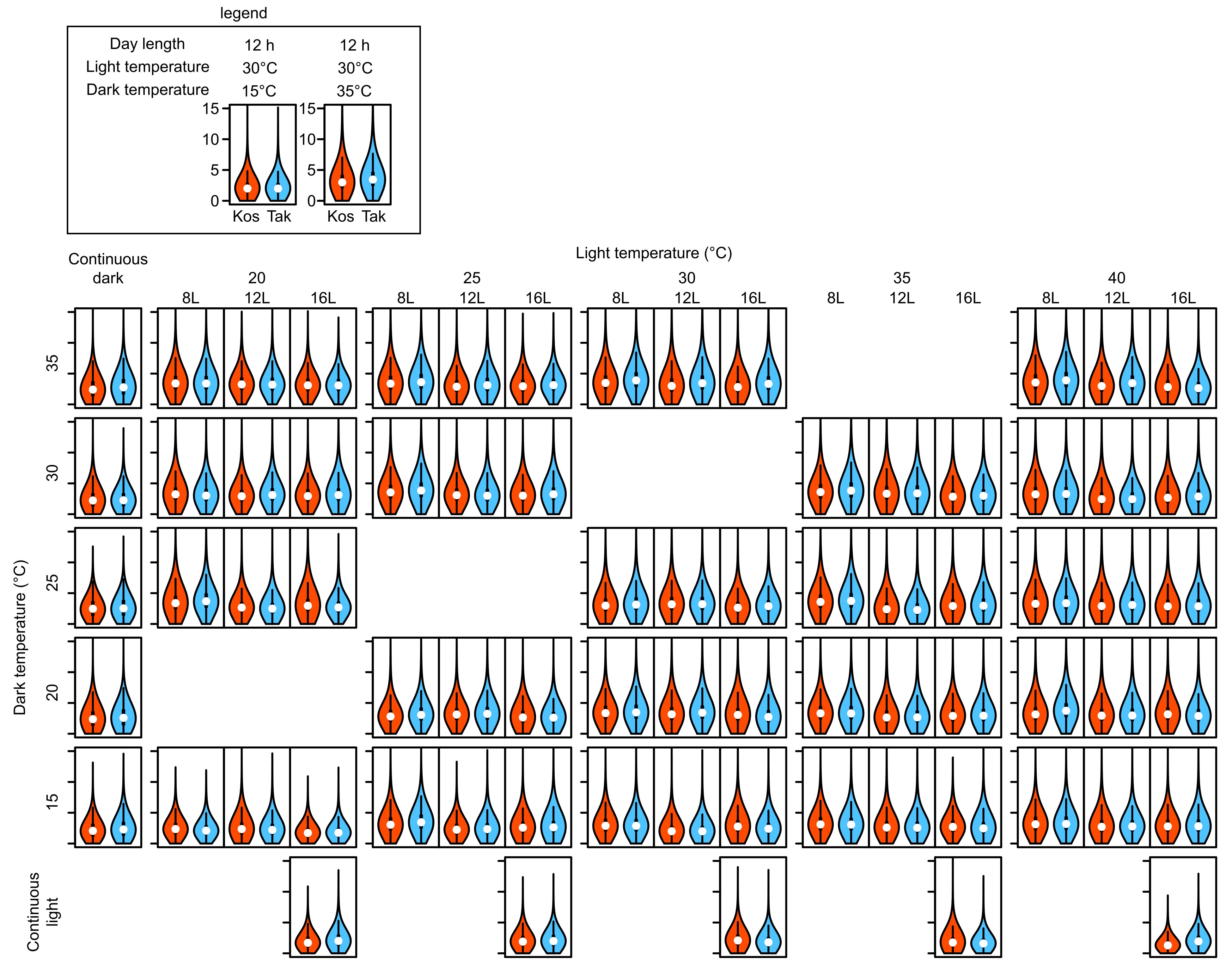
**Fig. S4 Distribution of expression amplitudes across 73 conditions.**

In each subpanel, the distribution of expression amplitude of Koshihikari and Takanari at each condition is shown by violin plot in red and blue, respectively. The subpanels are arranged according to the light/dark temperature. Within each group, the 8L/16D, 12L/12D, and 16L/8D photoperiod conditions are arranged from left to right, whereas the 0L/24D and 24L/0D conditions are displayed separately. The x-axis, y-axis, and their scale values in the subpanels are the same as those in the legend.


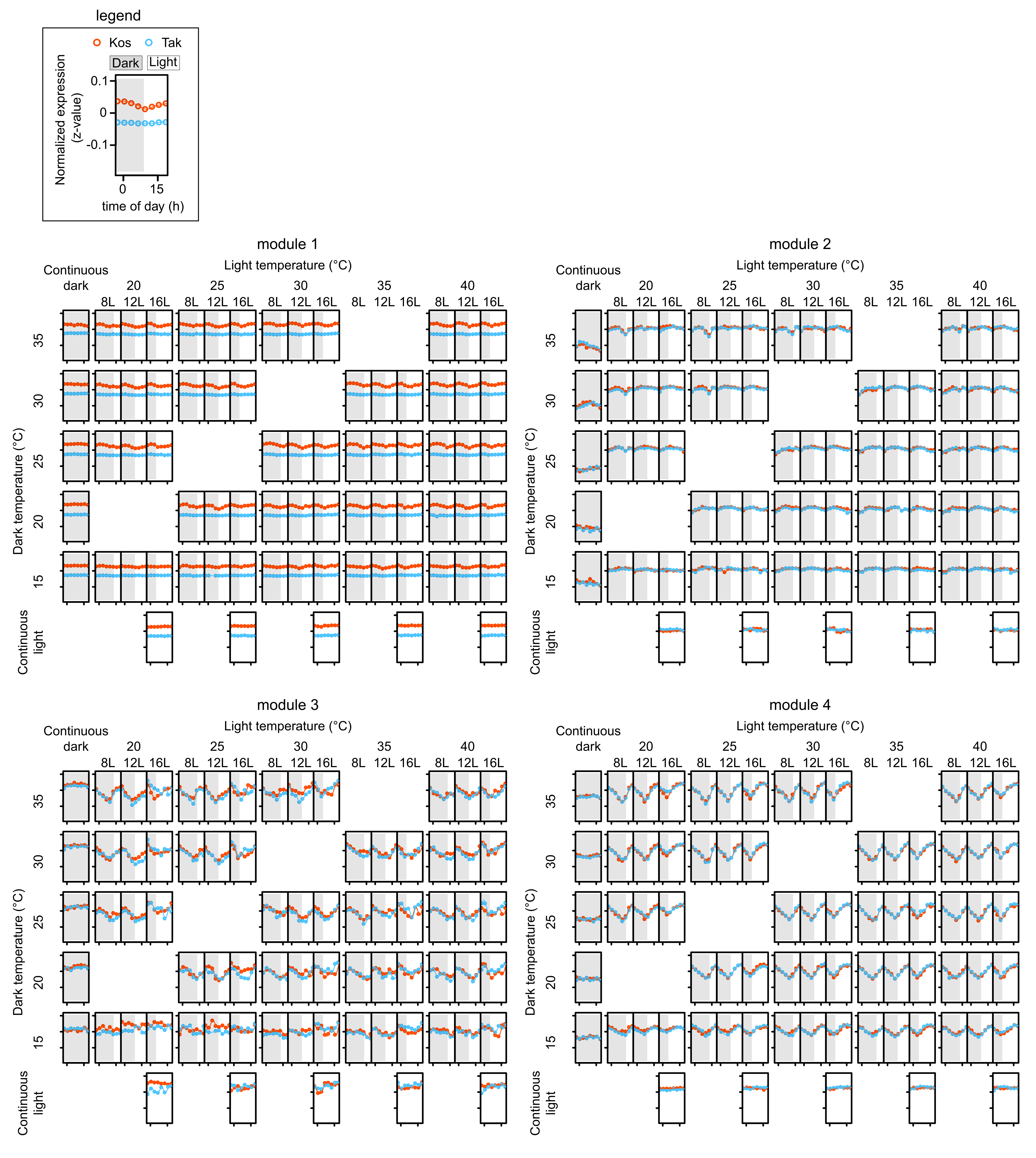


**
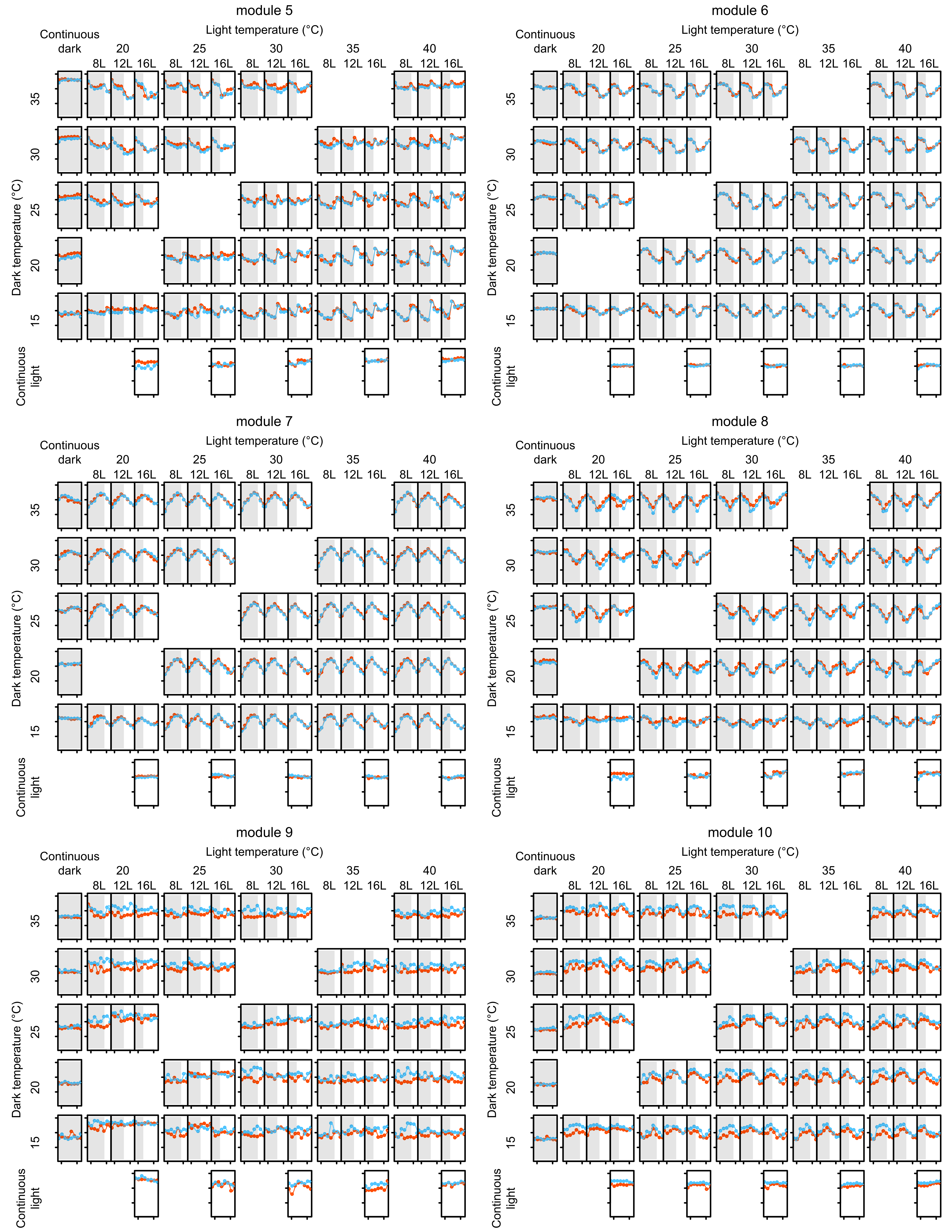
**

**
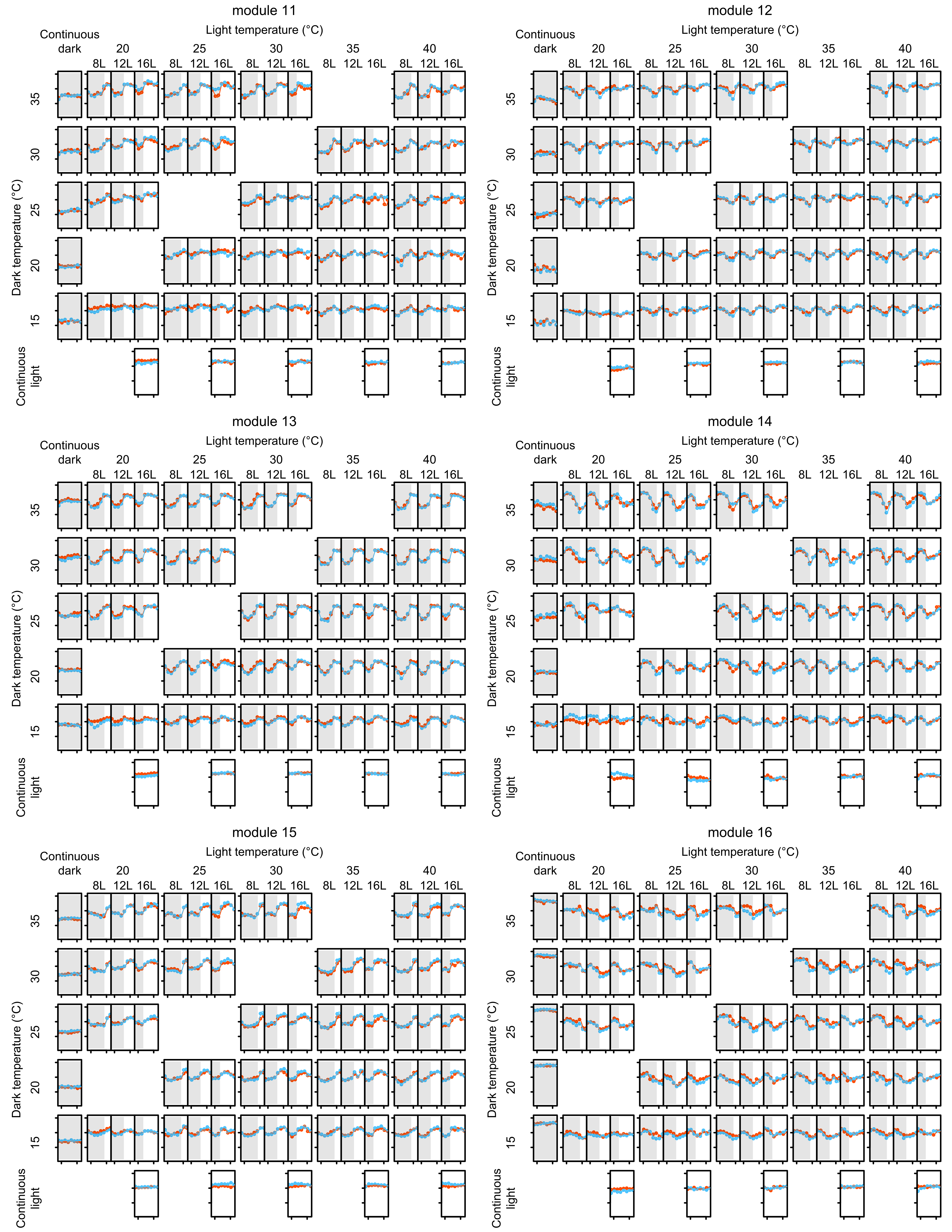
**

**
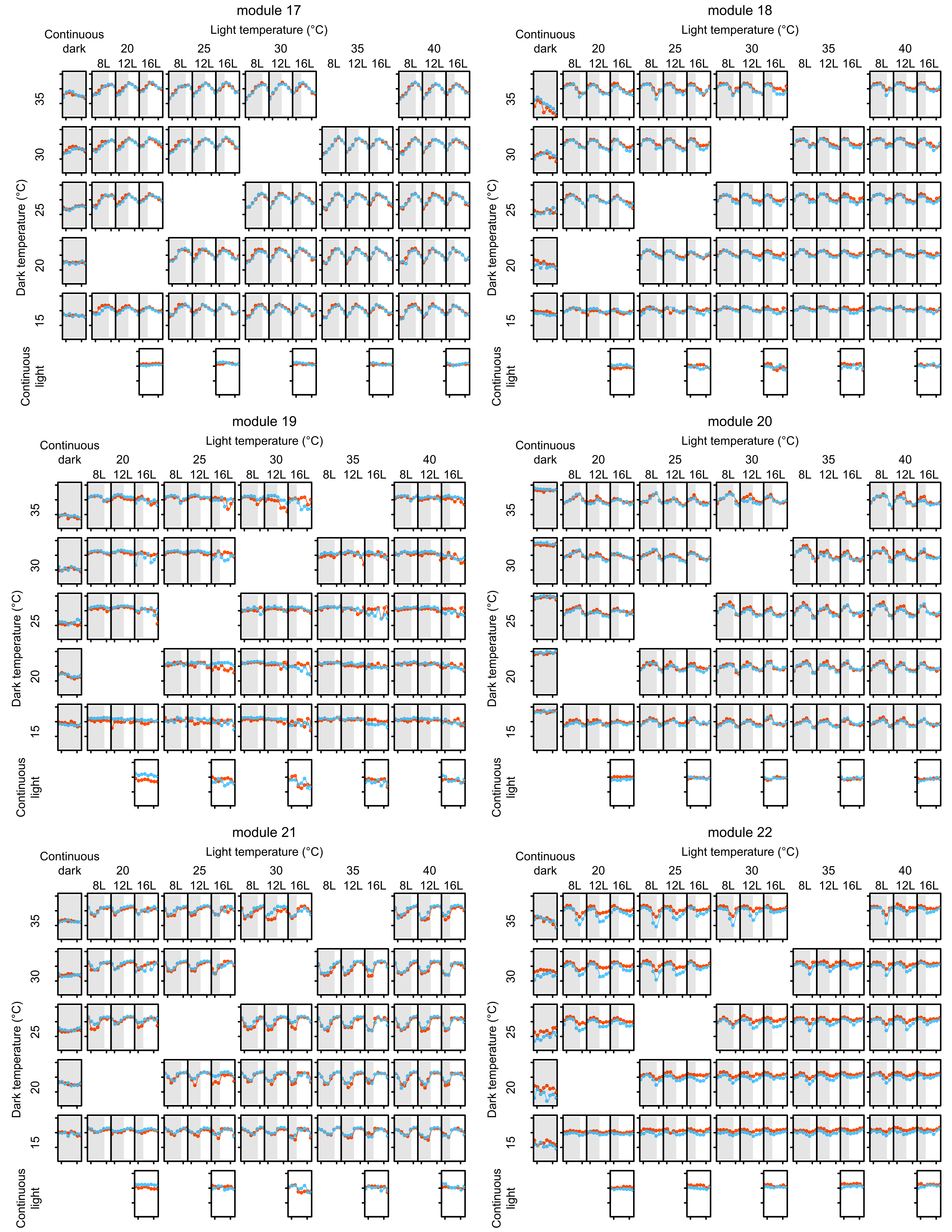
Fig. S5 Normalized expressions of eigengenes of each module.**

In each subpanel, normalized expression levels using z-scores of Koshihikari and Takanari at each condition are shown in red and blue, respectively. White and grey zones indicate light and dark periods, respectively. The subpanels are arranged according to the light/dark temperature. Within each group, the 8L/16D, 12L/12D, and 16L/8D photoperiod conditions are arranged from left to right, whereas the 0L/24D and 24L/0D conditions are displayed separately. The x-axis, y-axis, and their scale values in the subpanels are the same as those in the legend.

**
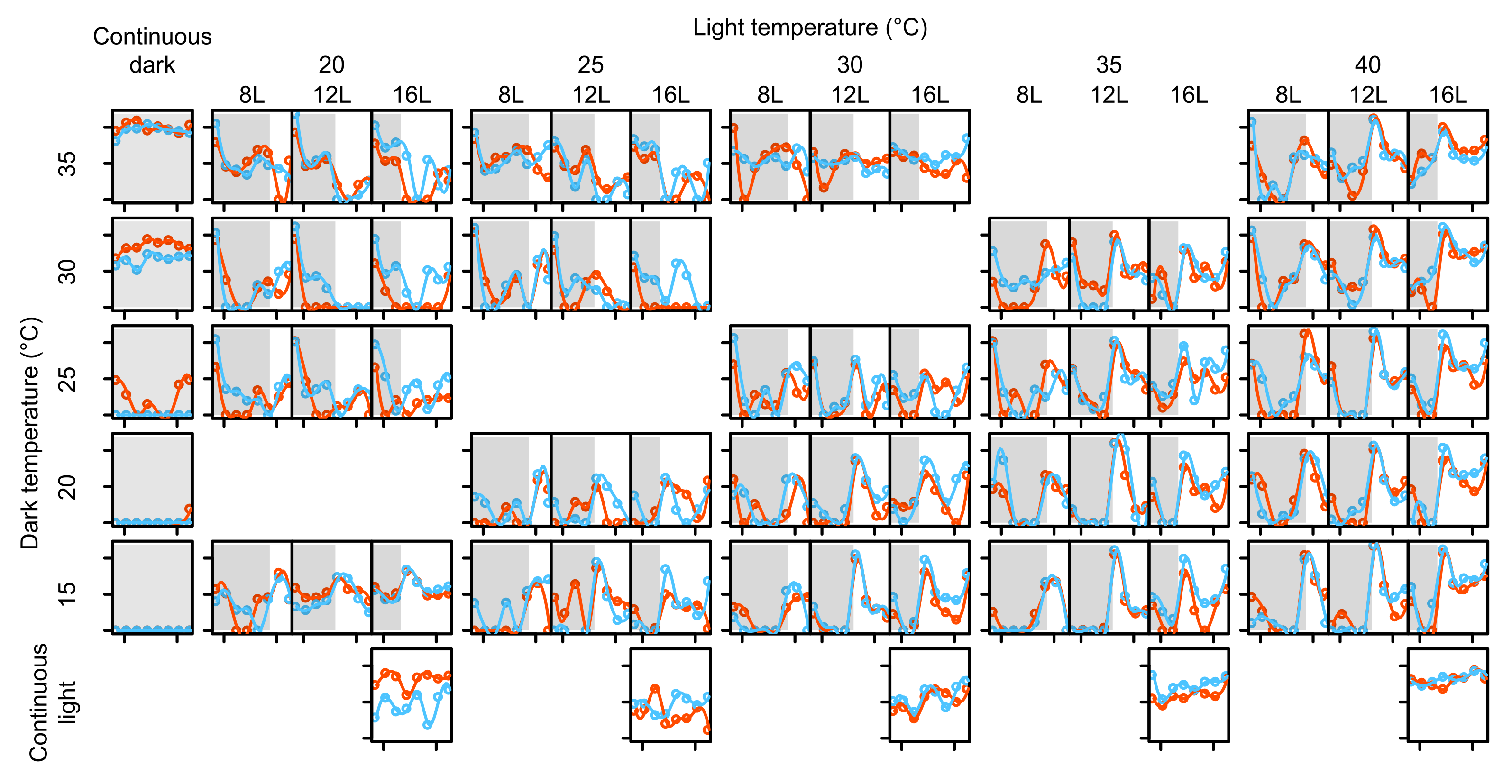
**

**Fig. S6 Expression levels of a gene encoding *Hsp 70* (*Os01g0840100*).**

In each subpanel, expression levels (log_2_(rpm+1)) of Koshihikari and Takanari at each condition are shown in red and blue, respectively. White and grey zones indicate light and dark periods, respectively. The subpanels are arranged according to the light/dark temperature. Within each group, the 8L/16D, 12L/12D, and 16L/8D photoperiod conditions are arranged from left to right, whereas the 0L/24D and 24L/0D conditions are displayed separately. The x-axis, y-axis, and their scale values in the subpanels are the same as those in the legend in Fig. 2a.

**
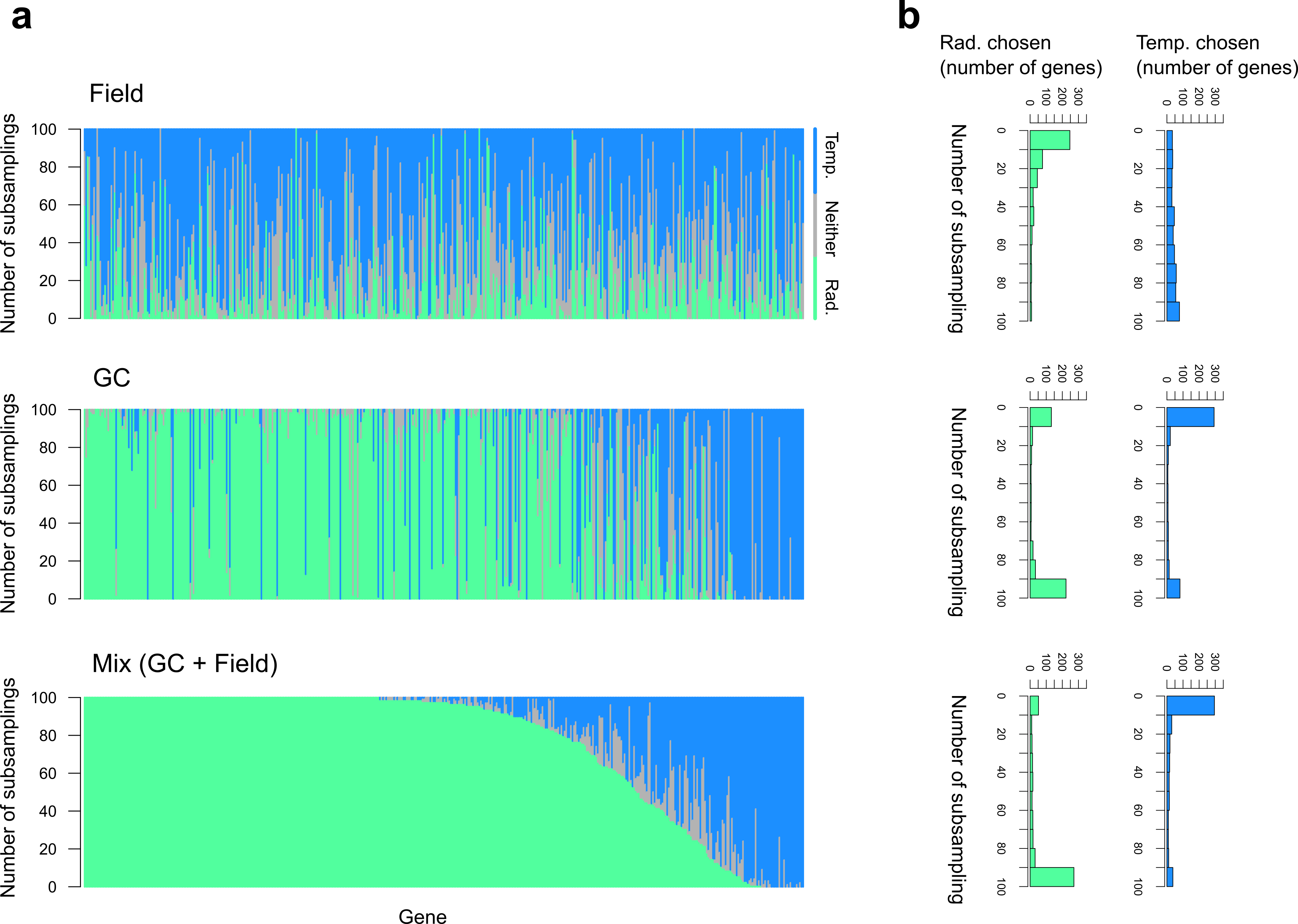
**

**Fig. S7 The number of times temperature or radiation (or neither) was chosen as the predictor of gene expression (Takanari).**

**a**, A single vertical line represents a gene whose green, blue, and grey parts show the frequency where radiation, temperature, or neither was chosen (among the 100 data subsampling). The 466 genes are sorted according to the frequency at which radiation was chosen in the model training with mixed (GC + field) data. **b**, Histograms showing how frequently temperature or radiation was chosen.

**
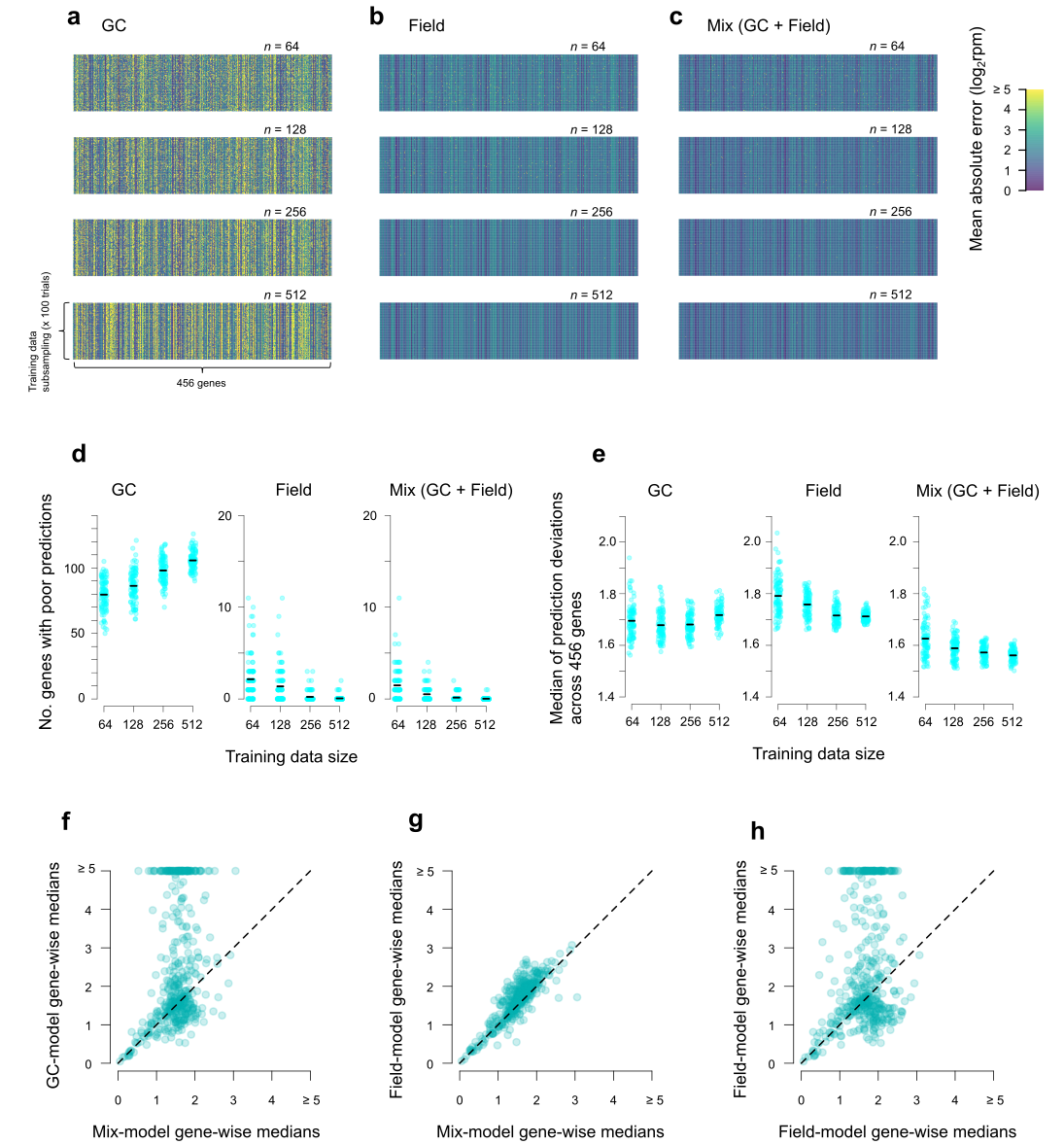
**

**Fig. S8 Prediction performances of gene expression models trained with different data sets in Takanari.**

The performances were evaluated by applying the models to the field test data (not included in the training data). **a**–**c**, Heat maps of mean absolute errors (MAE). The GC + Field (c) models were trained with a mixture of GC and field data (50% each). Training data size (*n*) was also varied. **d–h**, show summary statistics of **a–c**. **d**, The numbers of genes with poor predictions (log_2_rpm ≥ 5). **e**, Medians (across 456 genes) of the MAE values. In **d** and **e**, data points correspond to training data subsampling, and black horizontal lines represent means. **f–h**, Gene-wise medians of the MAE values compared between models trained with different data sets.

**
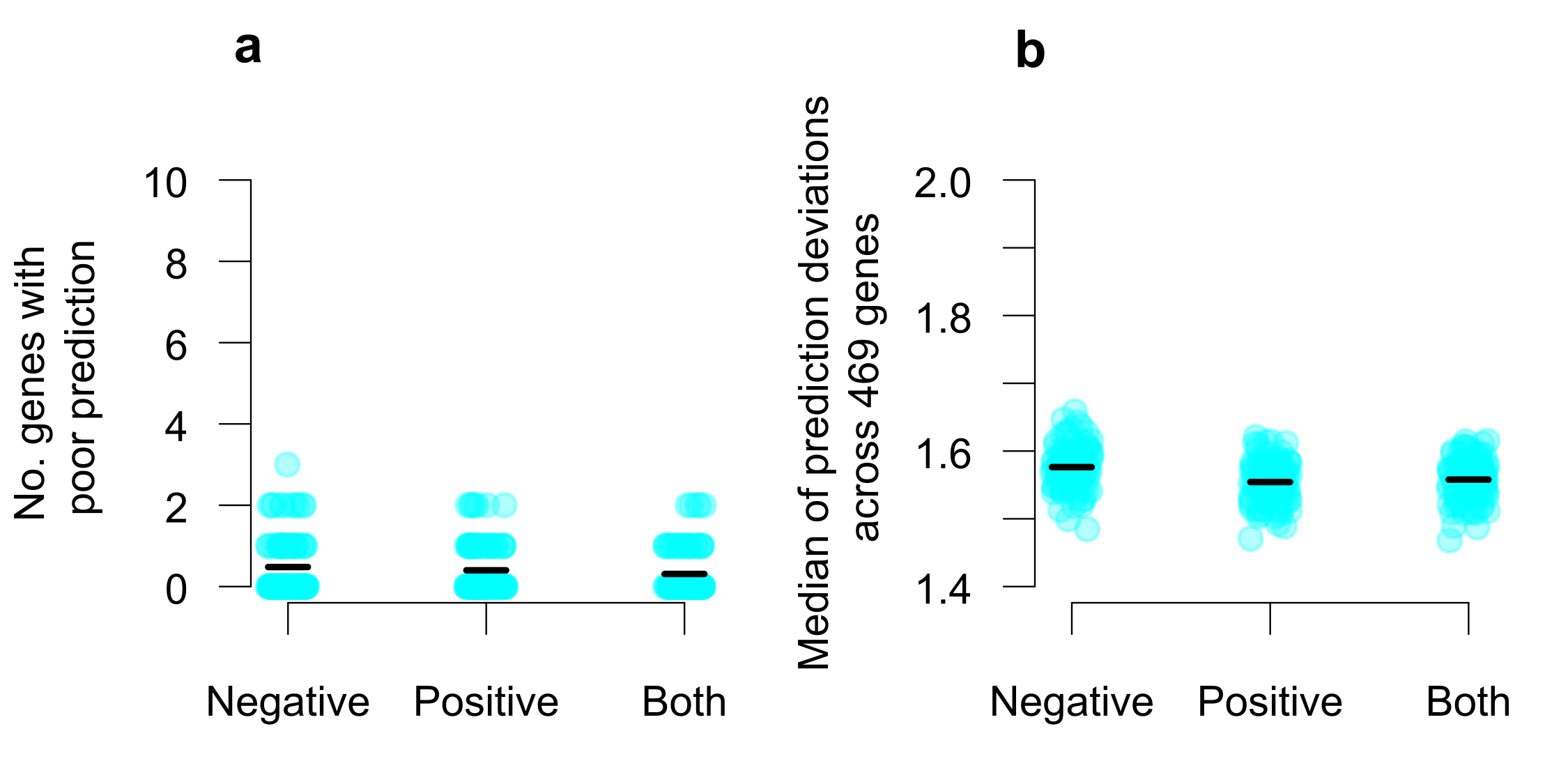
**

**Fig. S9 Prediction performances of gene expression models trained with different data sets in Koshihikari.** As in Fig. 5de, we trained models with the mixture of GC and field data (50% each), but this time we split the GC data into two conditions of positive or negative temperature-irradiance correlations. Negative: models that used only negatively correlated subsets of GC data. Positive: models that used only positively correlated subsets of GC data. Both: models that used both positively and negatively correlated subsets of GC data (50% each). The training data size is *n* = 288 (Field + GC) for all models.
